# NQO1‐Mediated Anoikis Resistance and Immune Evasion Define a High‐Risk Multi‐Omic Subtype for Precision Management of T1 High‐Grade Bladder Cancer

**DOI:** 10.1002/advs.202523605

**Published:** 2026-04-07

**Authors:** Bin Guo, Chunru Xu, Shufan Fu, Qiang Cheng, Juan Li, Linkuo Shang, Gaojie Li, Yang Yang, Ying Wang, Yanqing Gong, Shengwei Xiong, Jian Fan, Changwei Yuan, Mei Zhang, Yifan Zuo, Elena Papaleo, Yue Shi, Yuan Liang, Xuesong Li, Hongzhao Li, Weimin Ci

**Affiliations:** ^1^ China National Center for Bioinformation Beijing China; ^2^ Senior Department of Urology Chinese PLA General Hospital Beijing China; ^3^ Beijing Institute of Genomics Chinese Academy of Sciences Beijing China; ^4^ University of Chinese Academy of Sciences Beijing China; ^5^ Department of Urology Peking University First Hospital Beijing China; ^6^ Institute of Urology Peking University Beijing China; ^7^ National Urological Cancer Center Beijing Key Laboratory of Urogenital Diseases (Male) Molecular Diagnosis and Treatment Center Beijing China; ^8^ Chinese Institutes for Medical Research Beijing China; ^9^ Bioscience and Biomedical Engineering Thrust Systems Hub The Hong Kong University of Science and Technology (Guangzhou) Guangzhou Guangdong China; ^10^ Technical University of Denmark Department of Health and Technology Section for Bioinformatics Cancer Systems Biology Lyngby Denmark; ^11^ Danish Cancer Institute Cancer Structural Biology Copenhagen Denmark; ^12^ Institute for Regenerative Biology and Medicine Chinese Institutes for Medical Research Beijing China

**Keywords:** anoikis resistance, bladder‐preserving therapy, immune evasion, multi‐omics, NQO1, risk stratification, T1 high‐grade bladder cancer

## Abstract

T1 high‐grade (T1HG) bladder cancer represents an aggressive subset of non–muscle‐invasive bladder cancer (NMIBC) with frequent Bacillus Calmette–Guérin (BCG) failure and a high risk of progression, yet current models inadequately guide treatment selection between early cystectomy and bladder preservation. Integrative multi‐omics profiling of 147 tumors identifies two clinically distinct subtypes. A high‐risk subtype (T1HG1) is defined by coupled anoikis resistance and immune evasion, exhibiting markedly increased progression rates (>80% vs. <20%), poor BCG responsiveness, and a higher likelihood of cystectomy. NAD(P)H:quinone oxidoreductase 1 (NQO1) is identified as a central regulator linking tumor‐intrinsic survival to suppression of macrophage–T cell crosstalk. Elevated NQO1 promotes anoikis resistance and reprograms macrophages toward an immunosuppressive phenotype, limiting CXCL9‐mediated T cell recruitment and facilitating immune escape. Pharmacologic inhibition of NQO1 using skullcapflavone II restores apoptotic sensitivity and enhances cisplatin efficacy, resulting in significant tumor suppression with favorable tolerability in preclinical models. A multi‐omic machine learning framework for T1HG UCB, termed T1HG‐UCBguider, integrating clinical, transcriptomic, and methylation features, enables individualized risk stratification and treatment guidance. Validation across seven independent cohorts, demonstrates robust performance in identifying patients at risk of progression and BCG failure. These findings establish a biologically grounded framework for precision management of T1HG bladder cancer.

## Graphical abstract

Multi‐omic profiling of T1 high‐grade bladder cancer identifies a high‐risk subtype (T1HG1) driven by NQO1, which couples anoikis resistance with immune evasion. NQO1 orchestrates macrophage–T cell crosstalk suppression via CXCL9 modulation. Pharmacological NQO1 inhibition with skullcapflavone II enhances cisplatin efficacy, representing a promising bladder‐preserving strategy. The T1HG‐UCBguider framework enables precise risk stratification and treatment guidance.

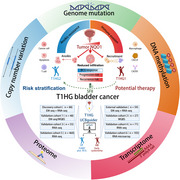

## 1 Introduction

Urothelial carcinoma of the bladder (UCB) is the second most common genitourinary malignancy worldwide [1]. It imposes a substantial economic burden, with annual treatment costs exceeding $4.25 billion in the United States and £55.39 million in the United Kingdom [2]. Among UCB cases, non‐muscle‐invasive bladder cancer (NMIBC) accounts for approximately 75%. T1 high‐grade (T1HG) tumors represent the highest‐risk subset, comprising ∼20% of NMIBC and ∼86 000 cases annually worldwide [3]. Although classified as non‐invasive, T1HG UCB shares biological features with muscle‐invasive disease, including frequent understaging of occult pT2+ lesions and a strong propensity for rapid progression [4]. After transurethral resection of bladder tumor (TURBT), recurrence rates range from 69% to 80%, and 33%–48% of patients progress to muscle‐invasive or metastatic disease [4]. These outcomes highlight the limitations of current risk stratification and treatment strategies.

Clinical management of T1HG UCB requires balancing treatment efficacy and quality of life [5, 6]. Early radical cystectomy (RC) provides the highest chance of cure for very‐high‐risk (VHR) patients but compromises quality of life [7]. In contrast, conservative approaches such as Bacillus Calmette–Guérin (BCG) immunotherapy may lead to undertreatment in high‐risk (HR) patients who will eventually progress [7]. Current prognostic models are largely based on clinicopathological features [7], including tumor size, multifocality, and concomitant carcinoma in situ. Although incorporated into clinical guidelines, these models show limited discriminatory ability in T1HG UCB [8, 9, 10, 11]. They often overestimate progression risk and fail to identify truly BCG‐refractory disease [11, 12, 13]. Recent molecular classifiers, including the published 23‐Gene Prognostic Index in 2024, have improved risk stratification in broader NMIBC populations [14]. However, they remain insufficient for guiding timely cystectomy in T1HG patients who fail or are resistant to BCG therapy. This gap represents a critical unmet clinical need [15].

There is an urgent need for biologically grounded, multi‐omic approaches that capture the intrinsic heterogeneity of T1HG UCB [7, 16, 17]. Emerging evidence suggests that molecular subtypes may better reflect tumor aggressiveness than histology alone [7, 18, 19]. However, existing classification systems, such as TCGA‐BLCA and UROMOL, were primarily derived from muscle‐invasive or mixed‐stage cohorts and lack resolution for T1HG‐specific biology [20, 21, 22, 23, 24]. In addition, the mechanisms underlying BCG resistance and disease progression in this subgroup remain poorly understood [25, 26]. In particular, the interaction between tumor‐intrinsic survival programs and the immunosuppressive tumor microenvironment requires further clarification.

Here, we define the molecular architecture of T1HG UCB through integrative multi‐omics and identify a high‐risk subtype (T1HG1) marked by coupled anoikis resistance and immune evasion that underlies BCG failure and disease progression. Mechanistically, we position NQO1 as a central node linking tumor‐intrinsic survival to suppression of macrophage–T cell crosstalk, thereby coordinating both metastatic competence and immune escape. Therapeutically, NQO1 inhibition restores apoptosis and enhances cisplatin sensitivity, supporting a rational bladder‐preserving combination strategy. Translating these insights, we develop T1HG‐UCBguider (a multi‐omic machine learning‐based predictor for T1HG UCB), a multi‐omic machine learning framework that enables precise identification of patients requiring early cystectomy while expanding candidates for conservative treatment. Cross‐cohort and urine‐based validation further underscores its potential for non‐invasive, precision management of T1HG UCB.

## 2 Results

### 2.1 Multi‐Omic Subtypes Define Clinically Distinct T1HG Bladder Cancer

**FIGURE 1:**
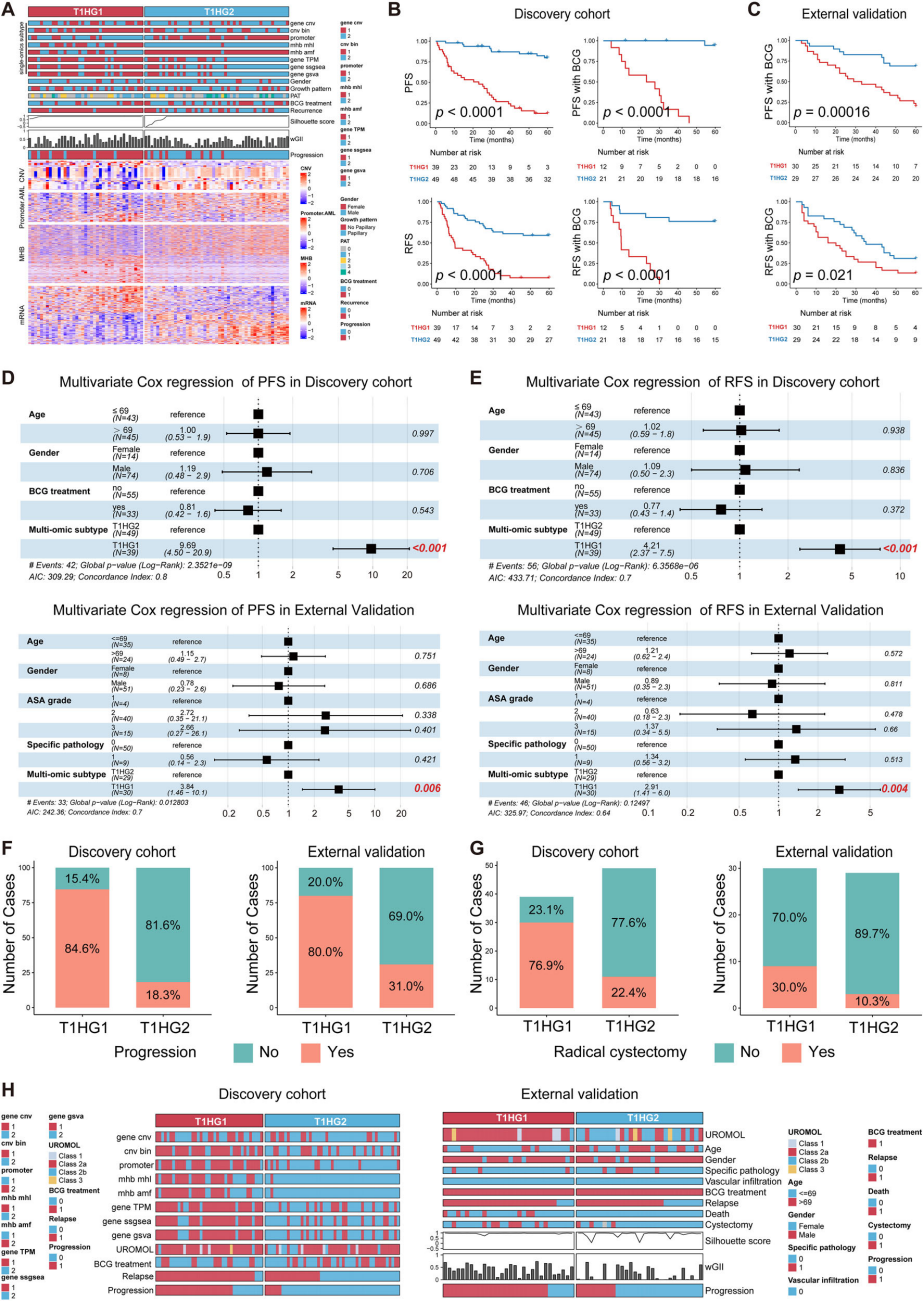
Identification and validation of a clinically relevant multi‐omic classification in T1 high‐grade bladder cancer. (A) Heatmap depicting the multi‐omic subtype stratified by the two molecular subtypes (T1HG1 and T1HG2) using integrated prognostic features (607 CNV genes, 156 promoters, 1505 MHBs, 284 mRNA features), along with clinicopathological features. The panel shows the correlation between molecular subtypes and clinical variables. (B,C) Kaplan–Meier curves showing progression‐free survival (PFS) and recurrence‐free survival (RFS) in the discovery cohort (B) and the external validation cohort (C), stratified by T1HG1 and T1HG2 subtypes. Both PFS and RFS were significantly worse in T1HG1 compared to T1HG2 in all T1 high‐grade patients and in the subset receiving BCG treatment. (D,E) Multivariate Cox regression analyses of PFS and RFS in the discovery cohort (D) and the external validation cohort (E). The T1HG1 subtype was independently associated with poor PFS and RFS, after adjusting for age, gender, BCG treatment, and other clinical factors. (F,G) Bar plots illustrating the proportion of patients with progression (F) and radical cystectomy (G) in both discovery and external validation cohorts. T1HG1 showed higher rates of progression and radical cystectomy, confirming its aggressive clinical behavior. (H) Oncoprint illustrating the correlations among single‐omic, multi‐omic subtypes, UROMOL classifications, and clinical parameters in the discovery cohort (left) and the external validation cohort (right). T1HG1 (*n* = 39) vs. T1HG2 (*n* = 49) and its subset of BCG treatment (*n* = 12 vs. *n* = 21) in the discovery cohort (*n* = 88). T1HG1 (*n* = 30) vs. T1HG2 (*n* = 29) fully treated with BCG in the external validation cohort (*n* = 59). The log‐rank test was used to compare the Kaplan–Meier survival curves (B and C). Multivariate Cox regression analysis was performed to analyze the relative risk of progression and recurrence (D and E). A *p*‐value < 0.05 is considered statistically significant. Statistical analysis was carried out using R (v4.2.1).

To identify molecularly distinct subtypes of T1HG UCB, we first overcame technical challenges in sequencing FFPE samples (Figure S12). We then performed an integrated analysis of paired whole‐genome enzymatic methylation‐sequencing (EM‐seq) and bulk RNA‐seq data in a discovery cohort (*n* = 88), which was validated in an external validation cohort (*n* = 59). The two cohorts were comparable in age and gender distribution, both characterized by detailed prognostic information (Figure S1A). To ensure robustness, we applied multiple quantification strategies in each single‐omic analysis. Univariate Cox regression analysis identified features significantly associated with progression‐free survival (PFS) (*p* < 0.05) in the discovery cohort and its BCG‐treated subset (*n* = 33). Overlapping features across single‐omic subtypes were selected for multi‐omic consensus clustering with an average silhouette width of 0.88 (Figure S1B), delineating two clinically relevant molecular subtypes with distinct molecular landscapes (Figure 1A). In both the discovery (Figure 1B; Figure S1C) and external validation (Figure 1C; Figure S1D) cohort, the T1HG1 subtype was markedly associated with poor prognostic survival and worse BCG response (discovery cohort and its BCG‐treated subset, PFS: *p <* 0.0001, RFS: *p <* 0.0001; external validation, PFS: *p =* 0.00016, RFS: *p =* 0.021), indicating that the T1HG1 subtype defines a high‐risk and BCG‐refractory population.

The high‐risk T1HG1 subtype emerged as an independent predictor of recurrence and progression, irrespective of tumor stage or grade, as confirmed by multivariable analysis in both the discovery cohort (PFS: HR = 9.69, *p* < 0.001; RFS: HR = 4.21, *p* < 0.001; Figure 1D) and the external validation cohort (PFS: HR = 3.84, *p =* 0.006; RFS: HR = 2.91, *p =* 0.004; Figure 1E). Furthermore, cases with the T1HG1 subtype had a significantly higher rate of disease progression (discovery cohort: 84.6% vs. 18.3%, external validation: 80% vs. 31%; Figure 1F) compared to those with the T1HG2 subtype. Notably, T1HG1 patients were approximately three times more likely to undergo radical cystectomy at the 5‐year follow‐up (discovery cohort: 76.9% vs. 22.4%, external validation: 30% vs. 10.3%; Figure 1G), supporting early cystectomy in patients with the T1HG1 subtype.

Although the multi‐omic subtype was partially consistent with UROMOL classifications in identifying high‐risk subtypes, the latter primarily classified 76.13% and 14.77% of cases as malignant classes 2a/2b in the discovery cohort, leading to risk overestimation [23, 24]; thus, multi‐omic subtype offers higher resolution and precision, overcoming the limitation of single‐omic subtyping (Figure 1H). Our prognostic multi‐omic subtype represents the key characteristics of different T1HG tumors, and can effectively stratify risks and guide clinical treatment decisions.

### 2.2 Multi‐Omic Profiling Reveals Divergent Molecular Programs Linked to Progression and BCG Response

To elucidate the molecular basis underlying the prognostic divergence between T1HG1 and T1HG2 subtypes, we conducted an integrative multi‐omic analysis encompassing somatic mutations, copy number variation (CNV), DNA methylation, and gene expression.

**FIGURE 2:**
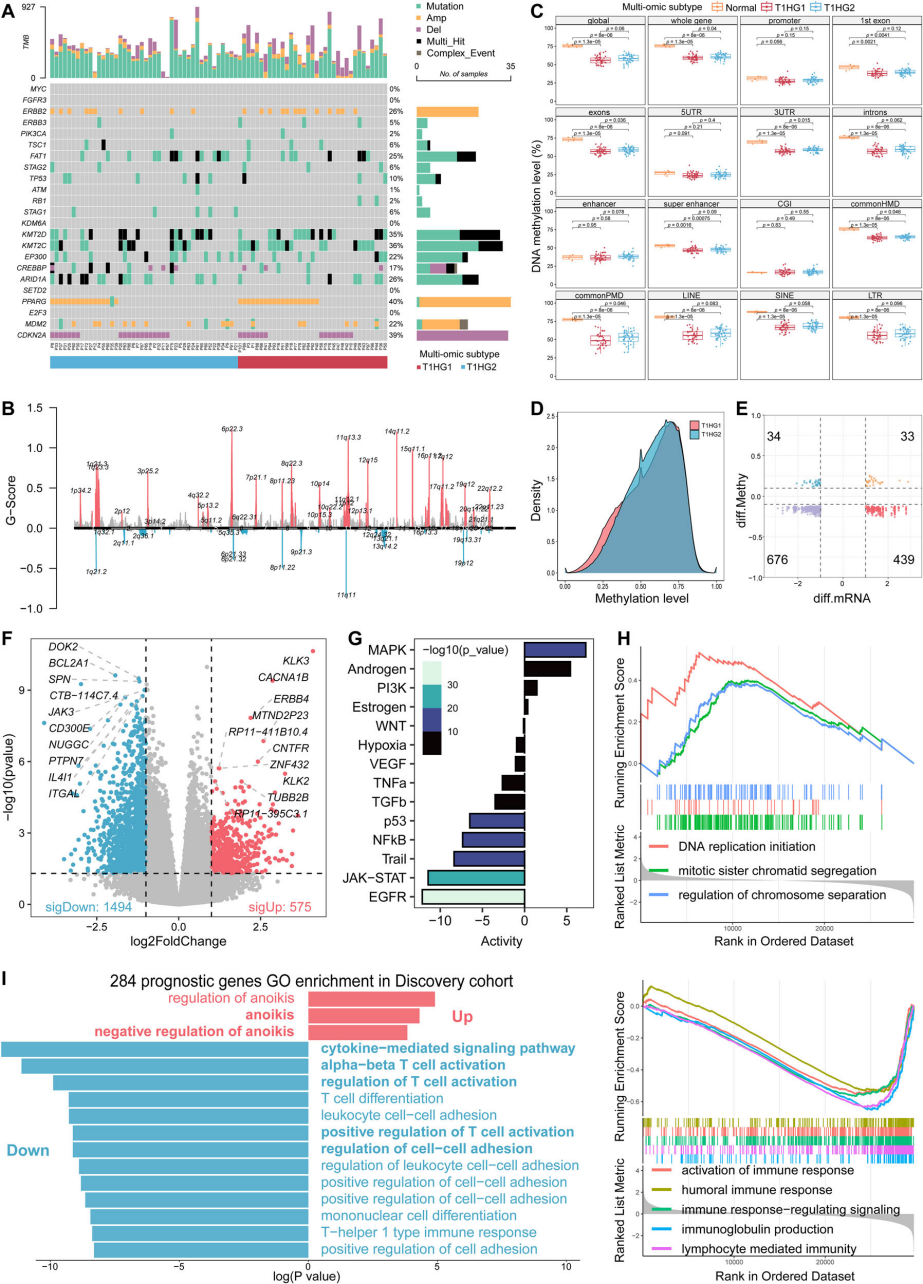
Multi‐omic profiling of T1 high‐grade bladder cancer reveals distinct molecular characteristics and functional pathways associated with cancer progression and immune response. (A) Integrated genomic landscape of key driver genes across samples stratified by multi‐omic subtypes (T1HG1 vs. T1HG2). Mutation (cyan), amplification (orange), deletion (purple), multi‐hit (dark gray), and complex events (light gray) are depicted. The bar plot on the right shows the frequency of each alteration type per gene, while the heatmap below displays the distribution of alterations across samples. Samples are colored according to their multi‐omic subtype (T1HG1: red; T1HG2: blue). (B) G‐score profile derived from copy number data highlighting significant differential copy number amplifications (red) and deletions (blue) between T1HG1 and T1HG2 subtypes. (C) Boxplots showing DNA methylation levels (averaged methylation rate) for selected CpG sites in key genomic regulatory elements across normal adjacent tissues and two multi‐omic subtypes (T1HG1 and T1HG2). (D) Density plot of DNA methylation levels across all CpG sites, comparing T1HG1 (red) and T1HG2 (green) subtypes. (E) Nine‐quadrant plot of differentially methylated regions (DMRs) and differentially expressed genes (DEGs) between T1HG1 and T1HG2. Red dots indicate significant differences, with 34 hypermethylated DMRs with lower expression and 439 hypomethylated DMRs with higher expression identified. (F) Volcano plot of DEGs in the discovery cohort. Genes significantly downregulated (sigDown: 1494) and upregulated (sigUp: 575) are highlighted in blue and red, respectively. The top significantly expressed DEGs are labeled. (G) Enrichment analysis of signaling pathways based on DEG activity scores. (H) Gene set enrichment analysis (GSEA) plots confirm significantly increased enrichment of cell cycle regulation and chromatin dynamics, and significantly reduced enrichment of immune‐related functions in the T1HG1 subtype. (I) Functional enrichment of 284 prognostic genes in the discovery cohort. Upregulated genes are enriched in anoikis‐related processes, while downregulated genes are associated with immune response and T‐cell activation pathways. The p‐values were calculated using a two‐tailed unpaired Mann‐Whitney U test (C). Each data point represents an individual sample. A *p*‐value < 0.05 is considered statistically significant. Statistical analysis was carried out using R (v4.2.1).

We first examined the mutational profile across all samples. The majority of somatic mutations were C>T transitions (Figure S2A), consistent with APOBEC activity [27, 28, 29]. No *FGFR3* mutation was detected (Figure 2A), which is consistent with the relatively low frequency of *FGFR3* mutations in Chinese bladder cancer patients [29, 30]. Meanwhile, no significant difference in overall mutation burden was observed between T1HG1 and T1HG2 (Figure 2A), indicating that tumor aggressiveness may not be driven by mutational load. Instead, genomic architecture differs markedly. CNV analysis detected a median of 28 amplifications and 23 deletions per sample, highlighting significant differences in copy number patterns between subtypes (Figure 2B; Figure S2B), and T1HG1 exhibited higher weighted genome instability index (wGII) scores (Figure S2C), suggesting greater genomic chaos despite similar mutation rates. GISTIC2.0 analysis of T1HG bladder cancer revealed recurrent amplifications at 17q12/*ERBB2* (33.3% vs. 20.4%), 3p25.2/*PPARG* (48.7% vs. 30.6%), and 12q15/*MDM2* (15.4% vs. 22.4%), as well as deletions at 9p21.3/*CDKN2A* (38.5% vs. 38.7%) in T1HG1 and T1HG2, respectively (Figure 2A).

We next analyzed whole‐genome DNA methylation profiles. Compared with adjacent normal tissues, T1HG tumors exhibit globally reduced DNA methylation (Figure 2C). However, T1HG1 and T1HG2 show distinct methylation patterns, with significant hypomethylation at the gene level, exons, 3’UTR, common highly methylated domain (HMD), common partially methylated domain (PMD) regions (Figure 2C). Meanwhile, T1HG1 exhibits more 10 kb genomic bins with average methylation levels between 0‐0.33 in density distribution (Figure 2D). Among 1505 prognostic methylation haplotype blocks (MHBs), 15.7% were located in promoter regions (< 2 kb) and 3.1% in exon regions (Figure S2D). Hypomethylated MHBs regions in T1HG1 are enriched for genes involved in cell cycle, chromatin organization, and developmental processes (e.g., *RB1*, *AKT2*, *MDM2*, *SOX2*, *CDKN2D*, *KRT5*), consistent with a proliferative phenotype (Figure S2E). In contrast, hypermethylated MHBs regions in T1HG1 are enriched for immune response and metabolic pathway genes, including *BIRC2*, *TRAF3*, *NR4A1*, *SOCS5*, *NFKBIZ*, indicating epigenetic silencing of anti‐tumor immunity (Figure S2E). Strikingly, differentially methylated regions (DMRs) correlate strongly with gene expression changes: 34 hypermethylated DMRs are associated with gene downregulation, and 439 hypomethylated DMRs with gene upregulation (Figure 2E). These results indicate that methylation‐driven regulation shapes subtype‐specific biology.

Gene expression profiling revealed 1494 downregulated and 575 upregulated differentially expressed genes (DEGs) in T1HG1 (Figure 2F). Upregulated genes in T1HG1 are enriched in developmental pathways, whereas downregulated genes are significantly enriched in immune‐related functions, consistent with DNA methylation profiles and confirming immune suppression (Figure S2F). Pathway activity analysis further supports this dichotomy. T1HG1 shows significant activation of MAPK, PI3K/AKT, WNT, and androgen receptor signaling pathways, while suppressing hypoxia, NFκB, JAK‐STAT, and EGFR signaling (Figure 2G). Notably, T1HG1 tumors display impaired DNA damage response (DDR), with significant downregulation of base excision repair (BER) genes such as *PARP3* and *CCNO* (Figure S2G). Gene set enrichment analysis (GSEA) confirms that T1HG1 is enriched in cell cycle and chromatin remodeling, while T1HG2 shows stronger enrichment in immune responses (Figure 2H). Furthermore, the GO enrichment of 284 prognostic genes indicated that T1HG1 tumors are enriched in genes promoting resistance to anoikis (loss of anchorage‐induced apoptosis [31, 32, 33]) and downregulation in cytokine signaling, T‐cell activation, and immune‐related pathways (Figure 2I), suggesting that anoikis resistance and immune evasion drive disease progression and BCG resistance of T1HG UCB.

Overall, our findings reveal that T1HG1 and T1HG2 subtypes are defined by fundamentally different biological programs involving genomic stability, epigenetic reprogramming, and transcriptional state remodeling.

### 2.3 T1HG1 Subtype Defines an Immune Evasive State with Reduced Macrophage T Cell Crosstalk

To define the immune landscape underlying the distinct clinical behaviors of T1HG1 and T1HG2 subtypes, we performed a comprehensive analysis of the tumor microenvironment (TME) using gene expression signatures, deconvolution algorithms, single‐cell RNA sequencing (scRNA‐seq), and multiplex immunofluorescence (mIF).

**FIGURE 3:**
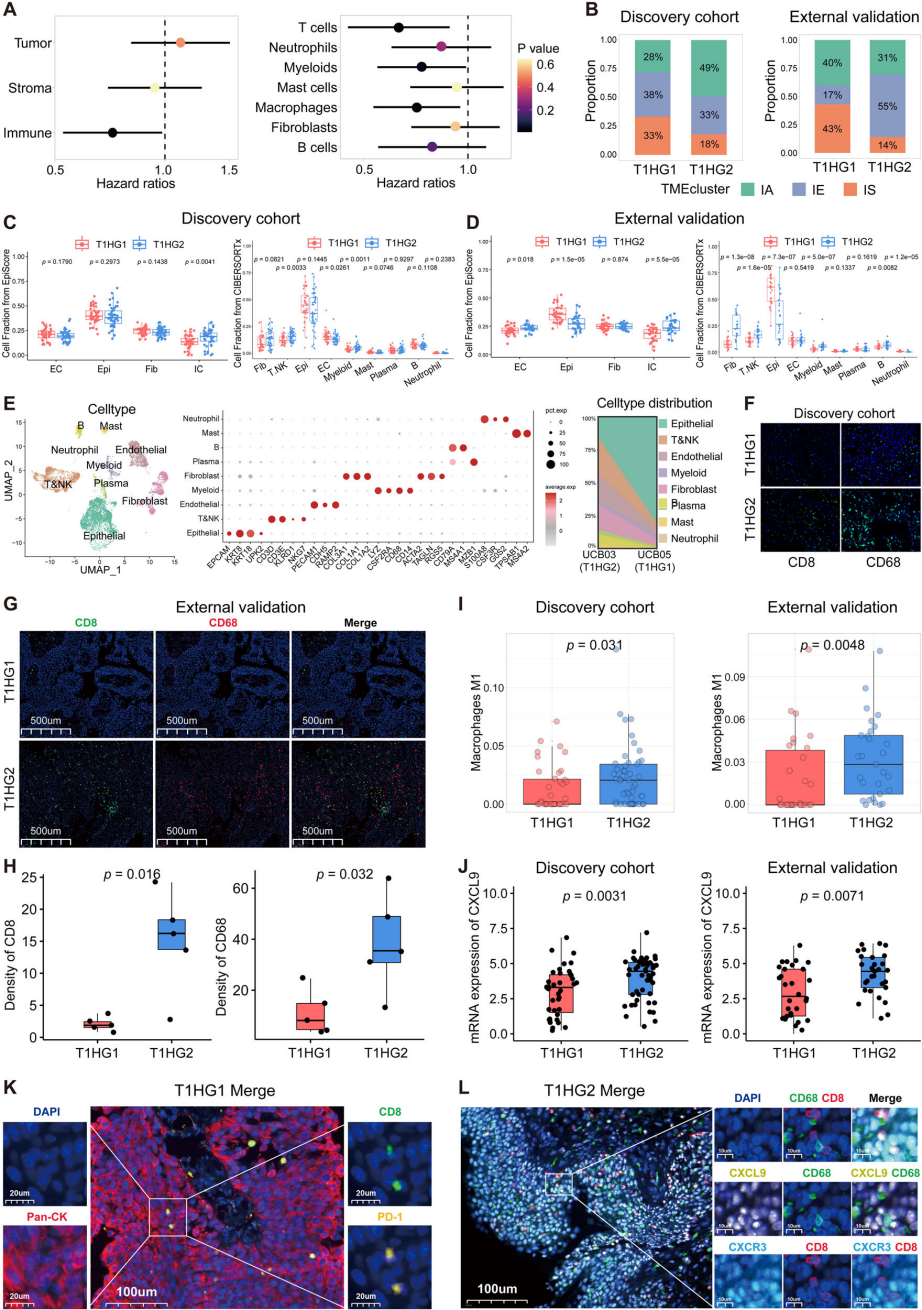
Immune microenvironment characterization reveals distinct immune infiltration patterns and spatial immune signatures associated with T1 high‐grade bladder cancer subtypes. (A) Comparison of tumor microenvironment‐derived prognostic signatures in the discovery cohort: gene expression signatures generated using pan‐compartment (tumor/stromal/immune) markers (left) and immune cell type‐specific markers (right). Multivariable Cox proportional hazards regression quantified the association strength (hazard ratios with 95% CI). (B) Proportion of TME clusters (Immune Active: IA, Immune Excluded: IE, Immune Suppressed: IS) in the discovery cohort (left) and external validation cohort (right), stratified by multi‐omic subtypes (T1HG1 and T1HG2). (C,D) Cellular deconvolution analysis of the discovery cohort (C) and the external validation cohort (D) using EPISCORE with EM‐seq methylation data (left) and CIBERSORTx with RNA‐seq transcriptome data (right). (E) UMAP plot of single‐cell transcriptomic data from two T1HG UCB tissues showing nine major cell types (left). A dot plot shows the expression levels of marker genes across cell types (middle). Comparison of cell proportion distribution in T1HG patients from two different subtypes (right). (F) Representative immunofluorescence (IF) images of CD8 and CD68 staining in the discovery cohort (*n* = 3 patients per subtype). (G,H) Representative multiplex IF images (G) of tumor‐infiltrating CD8^+^ T cells and macrophages (DAPI: blue, CD8: green, CD68: red) in two subtypes from the external validation cohort (*n* = 5 patients per subtype). Quantification of IF density from multiplex IF panoramic scanning images (H). (I) Boxplot of macrophage M1 polarization status (derived from mRNA expression of M1‐related genes) in the discovery and external validation cohort. (J) mRNA expression levels of *CXCL9* in the discovery and external validation cohorts. (K) Representative multiplex IF staining image of a T1HG1 tumor showing CD8^+^ T cells (green), PD‐1 (yellow), Pan‐CK (red), and DAPI (blue) in the tumor region from the external validation cohort (*n* = 3 patients per subtype). (L) Representative multiplex IF staining images of a T1HG2 tumors showing CD68^+^ macrophages (green), CXCL9 (yellow), CD8^+^ T cells (red), CXCR3 (cyan), and DAPI (blue) in the tumor region from the external validation cohort (*n* = 3 patients per subtype). Univariate Cox regression analyses were performed to assess the relative risk of signatures (A). The p‐values were calculated using a two‐tailed unpaired Mann‐Whitney U test (C, D, H, I, and J). Each data point represents an individual sample. A *p*‐value < 0.05 is considered statistically significant. Statistical analysis was carried out using R (v4.2.1).

In the tumor microenvironment, immune cells are the predominant components influencing the progression of T1HG UCB, and tumor‐infiltrating immune cells, including T cells and macrophages, are differentially associated with survival outcomes across subtypes (Figure 3A). Notably, T‐cell‐related genes (e.g., *FASLG*, *TNFRSF18*) and macrophage‐related genes (e.g., *CXCL9*, *CCL13*) were significantly associated with favorable outcomes (Figure S3A). Using a validated TME classification system [34, 35], we stratified tumors into three immune contexts. T1HG1 exhibited elevated proportions of immunosuppression (discovery cohort: 33% vs. 18%; external validation: 43% vs. 14%; Figure 3B). This indicates that T1HG1 exhibits a strongly immunosuppressive phenotype, whereas T1HG2 maintains immune engagement, despite some exclusion (Figure 3B). Cellular deconvolution analyses using EPISCORE [36] (methylation‐based) and CIBERSORTx [37] (transcriptome‐based) consistently demonstrated that T1HG1 tumors contain significantly higher proportions of malignant epithelial cells and lower levels of immune cells, including T/NK and myeloid cells compared to T1HG2 (Figure 3C). These findings were replicated in the external validation cohort (Figure 3D), confirming robustness across platforms and cohorts. scRNA‐seq analysis of two T1HG tumors identified nine major cell types, and cell‐type proportion analysis revealed enrichment of malignant epithelial cells and depletion of immune populations in T1HG1‐like tumors, consistent with bulk deconvolution results (Figure 3E; Figure S3B).

Representative mIF images from the discovery (Figure 3F) and external validation (Figure 3G) cohorts showed significantly lower densities of CD8^+^ T cells (*p =* 0.016) and CD68^+^ macrophages (*p =* 0.032) in T1HG1 tumors (Figure 3H). Moreover, T1HG1 tumors exhibited reduced M1‐like macrophage activity (*p =* 0.031 and *p =* 0.048 in the discovery and external validation cohort, respectively; Figure 3I; Figure S3C) and lower expression of *CXCL9*, a key chemokine of macrophage for T‐cell recruitment (*p =* 0.0031 and *p =* 0.0071; Figure 3J). These data suggest impaired antigen presentation and T‐cell trafficking in T1HG1. Multiplex IF staining also revealed co‐expression of CD8 and PD‐1 in T1HG1 tumors, indicating the presence of exhausted T cells within the tumor parenchyma (Figure 3K). In contrast, T1HG2 tumors display strong co‐localization of CD8^+^ T cells and CD68^+^ macrophages, with spatial proximity of CXCL9 (from macrophages) and CXCR3 (on T cells), suggesting functional immune cell crosstalk and chemokine‐mediated recruitment (Figure 3L). This indicates an active adaptive immune response in T1HG2 that is absent in T1HG1. Therefore, this impaired immune microenvironment likely contributes to the increased progression risk and suboptimal BCG therapy response observed in T1HG1‐subtype patients.

### 2.4 NQO1 Drives Anoikis Resistance and Tumor Progression in the Aggressive T1HG1 Subtype

**FIGURE 4:**
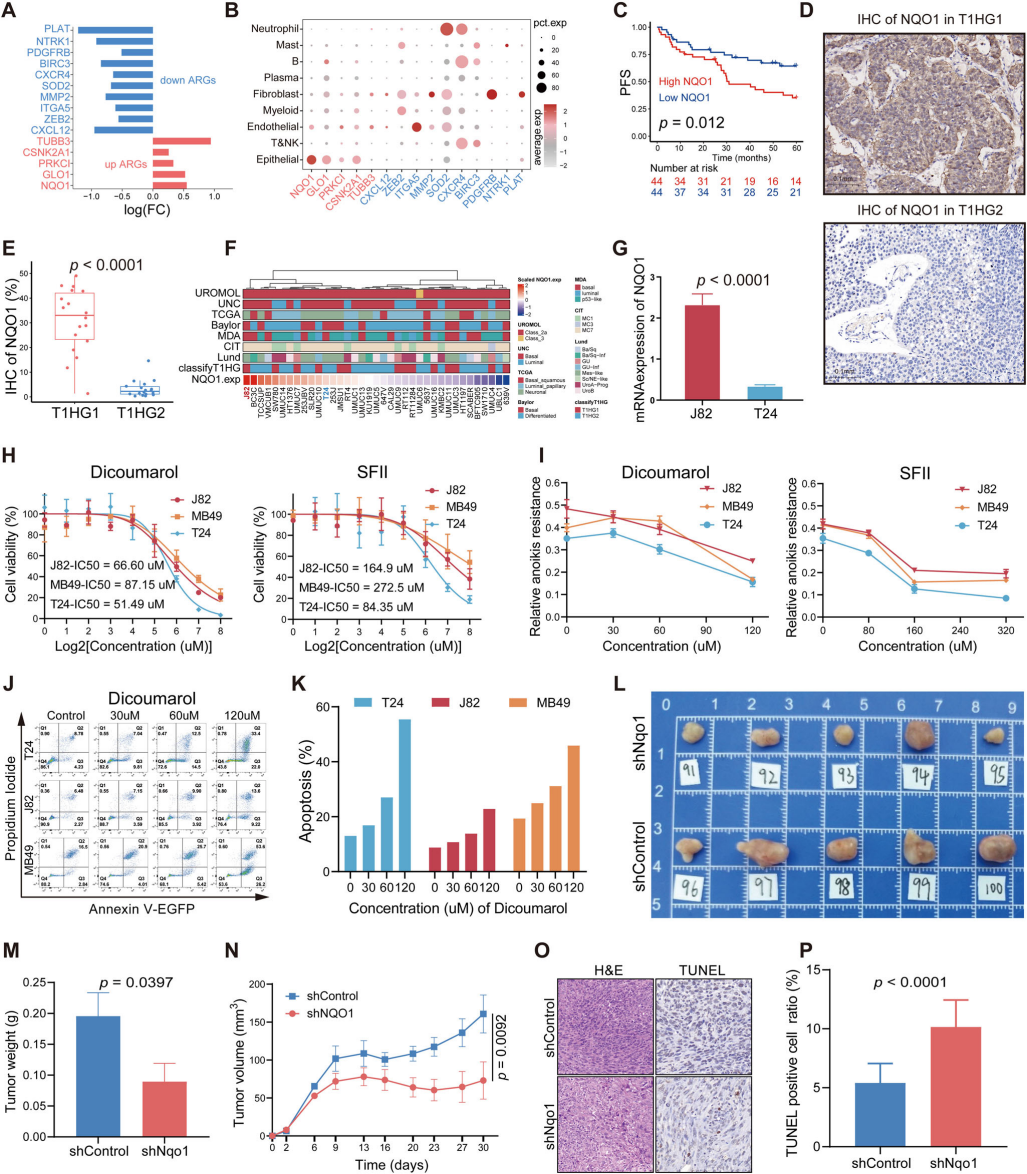
NQO1 serves as a key prognostic and therapeutic target in T1 high‐grade bladder cancer exhibiting differential expression and function across molecular subtypes. (A) Log_2_ fold change of anoikis‐related genes (ARGs) significantly upregulated or downregulated in the discovery cohort. (B) Dot plot showing gene expression levels (pct.exp and average expression) of *NQO1* across various cell types from single‐cell RNA‐seq data. (C) Kaplan–Meier survival curve showing progression‐free survival (PFS) stratified by high vs. low *NQO1* expression. (D,E) Representative immunohistochemistry (IHC) staining images of NQO1 protein in tumor samples from T1HG1 and T1HG2 (D). Boxplot of IHC‐positive area percentage of NQO1 in T1HG1 and T1HG2 cohorts (E), *n* = 18 patients for each subtype. (F) Canonical molecular subtypes and “classifyT1HG” classifications of UCB human cell lines based on public RNA‐seq datasets. (G) Bar plot of qPCR results verifying the relative mRNA expression of *NQO1* for J82 and T24, *n* = 4 replicates for each cell line. (H) The 50% inhibitory concentration (IC50) curves of NQO1 inhibitors, dicoumarol and Skullcapflavone II (SFII), in human cell lines (J82 and T24), and mouse cell line (MB49), *n* = 3 replicates for each cell line. (I) For the anoikis resistance assay, J82, T24, and MB49 cell lines were seeded onto non‐adherent plates for 24 h with the treatment of NQO1 inhibitors (dicoumarol and SFII), followed by using MTT Colorimetric Detection to quantify the relative anoikis resistance, *n* = 4 replicates for each cell line. (J,K) Flow cytometry analysis of apoptosis induction by dicoumarol at 30, 60, and 120 µm in T24, J82, and MB49 cells, *n* = 2 independent experiments. Annexin V‐EGFP/Propidium Iodide double staining reveals dose‐dependent increase in early and late apoptotic populations (J). Bar graph summarizing the percentage of apoptotic cells after dicoumarol treatment across concentrations and cell lines (K). (L–N) In vivo tumor growth kinetics in subcutaneous models (C57 male mice) using MB49 cells transduced with shControl or shNqo1, *n* = 5 C57 male mice per group. Knockdown of Nqo1 results in significantly reduced tumor volume and tumor weights at endpoint (L, M) and suppressed tumor volume over time (N). (O,P) Representative H&E and TUNEL staining images of tumors from shControl and shNqo1 groups (O). Quantitative analysis of TUNEL staining (P) showing significantly enhanced apoptosis in NQO1‐depleted tumors. shNqo1 tumors exhibit increased hypoxia and vascularization (H&E staining), and increased tumor apoptosis (TUNEL+ cells). The log‐rank test was used to compare the Kaplan–Meier survival curves (C). Data are shown as mean ± SEM. The *p*‐values were calculated using a two‐tailed unpaired Mann‐Whitney U test (E and P), two‐tailed unpaired Student's *t*‐test (G and M), and two‐way ANOVA followed by Tukey's post hoc test (N). A *p*‐value < 0.05 is considered statistically significant. Statistical analysis was carried out using R (v4.2.1) or GraphPad Prism (v8.0.2).

The aforementioned enrichment analysis of the 284 prognostic genes highlighted upregulated anoikis resistance in T1HG1 (Figure 2I), prompting further mechanistic investigation into adverse outcomes for T1HG UCB. As apoptosis is induced by loss of cell‐matrix contact, anoikis is a critical barrier to tumor muscle invasion [31, 32, 33]. We initially used 332 anoikis‐related genes (ARGs) from the previous study [38] to construct an ARGs score based on the ssGSEA algorithm. T1HG1 demonstrates significantly lower anoikis scores but higher tumor anoikis resistance (*p* < 0.001 in both cohorts; Figure S4A). In both discovery and external validation cohorts, differential expression analysis of ARGs identified five genes (*NQO1*, *GLO1*, *PRKCI*, *CSNK2A1*, and *TUBB3*) that were upregulated in tumor tissues, particularly in the T1HG1 subtype (Figure 4A; Figure S4B). Single‐cell RNA‐seq analysis confirmed strong *NQO1* expression in tumor epithelial cells (Figure 4B). Therefore, given its documented roles in tumorigenesis and progression in other cancers [39, 40], we investigated NQO1—a redox‐regulating enzyme implicated in stress response and chemoresistance. Kaplan–Meier analysis demonstrated that patients with high *NQO1* expression had significantly shorter progression‐free survival (PFS) (*p =* 0.012; Figure 4C). This finding was validated in the TCGA cohort, where high *NQO1* mRNA correlated with worse recurrence‐free survival (*p =* 0.035; Figure S4C). T1HG1 tumors exhibited significantly higher *NQO1* mRNA levels than T1HG2 (Figure 4A; Figure S4B,D), consistent with its association with aggressive biology. Immunohistochemistry confirmed intense NQO1 protein staining in T1HG1 tumors (*p* < 0.0001, Figure 4D,E), supporting translational relevance.

Subsequently, we investigated the therapeutic targeting of NQO1 at the cellular level using two human bladder cancer cell lines (J82 and T24) and a murine bladder cancer cell line (MB49). Importantly, J82 exhibited significantly higher *NQO1* expression compared to T24 (*p* < 0.001) with more malignant molecular subtypes (Figure 4F,G). Dicoumarol, a potent NQO1 inhibitor, is limited by its life‐threatening side effects as an anticoagulant and subsequent hemorrhagic complications [41]. In a recent study utilizing comprehensive 2D NQO1 biochromatography, a well‐tolerated natural compound—skullcapflavone II (SFII)—emerged as a novel NQO1 inhibitor [42]. We initially determined the half‐maximal inhibitory concentration (IC50) of dicoumarol and SFII in these cell lines (Figure 4H), indicating sensitivity differences across lines. Notably, J82, which displayed higher malignancy with higher expression of *NQO1*, had higher IC50 values compared to T24 (dicoumarol: 66.60 µm vs. 51.49 µm; SFII: 164.9 µm vs. 84.35 µm; Figure 4H). In anoikis resistance assays, treatment with dicoumarol or SFII significantly reduced relative anoikis resistance in all three cell lines (Figure 4I), showing that NQO1 inhibition impairs tumor cell survival under detachment conditions. Flow cytometry using Annexin V/PI staining also revealed that dicoumarol treatment dose‐dependently increased apoptotic populations in three cell lines, confirming NQO1‐mediated suppression of tumor apoptosis (Figure 4J,K).

To further validate the functional role of NQO1 in tumorigenesis in vivo, we generated murine MB49 cells stably expressing shRNA targeting Nqo1 (shNqo1) (Figure S4E). In the subcutaneous xenograft mice model, shNqo1 tumors grew significantly slower and were smaller in volume (*p* < 0.001) and weight (*p* < 0.05) compared to shControl (Figure 4L–N; Figure S4F). Histological analysis revealed increased apoptosis (TUNEL+ cells, *p* < 0.0001) and signs of hypoxia and vascular disruption in shNqo1 tumors (Figure 4O,P). These findings confirm that NQO1 is essential for tumor growth and survival, suggesting the therapeutic potential of NQO1 inhibitors for bladder‐preserving treatment of high‐risk T1HG1‐subtype bladder cancer.

### 2.5 NQO1 Inhibition Enhances Cisplatin Efficacy and Enables Bladder‐Preserving Therapy in T1HG1 Subtype

**FIGURE 5:**
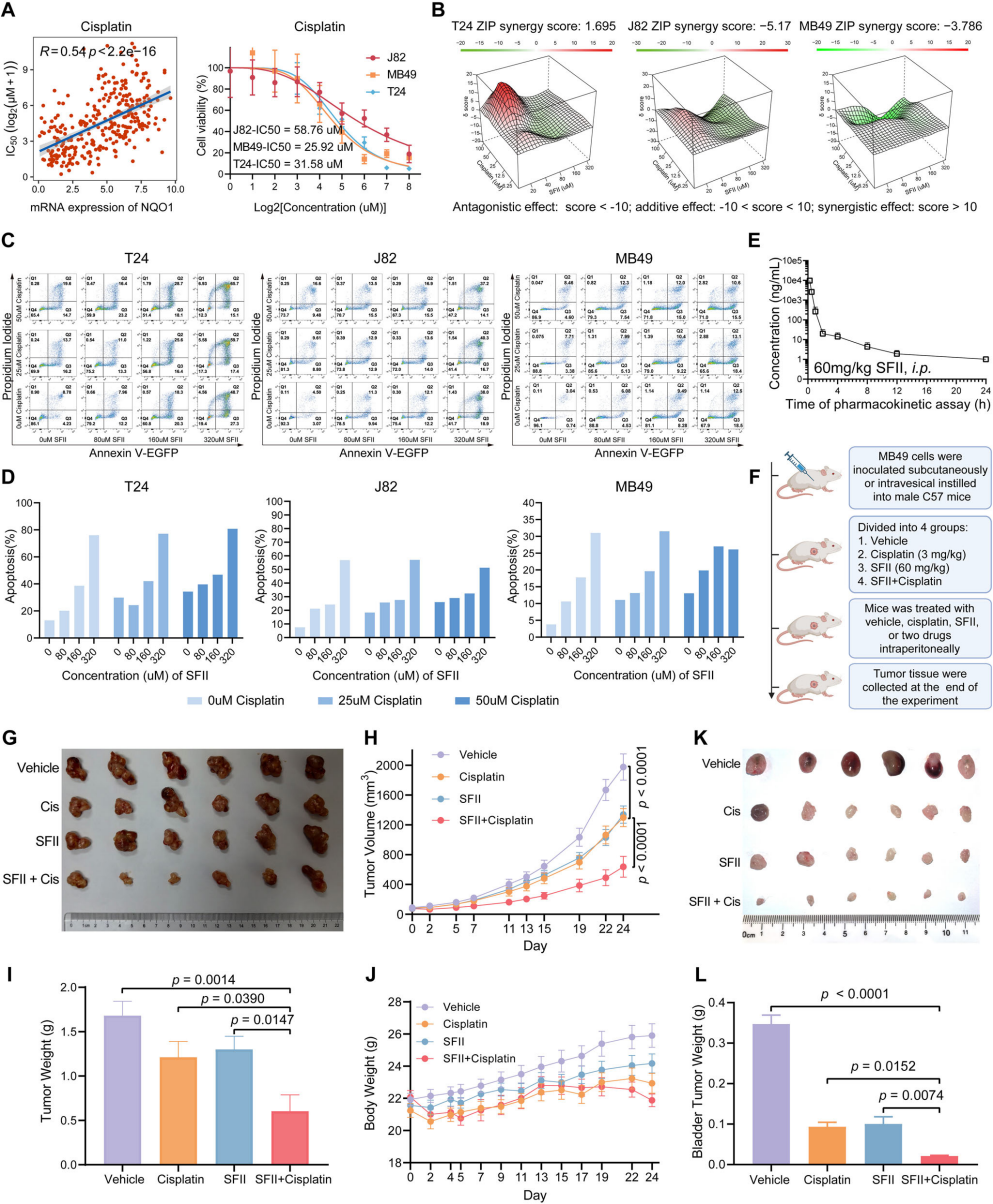
Skullcapflavone II enhances cisplatin efficacy through NQO1 inhibition as a potential bladder‐preserving treatment for high‐risk BCG‐refractory patients. (A) Scatter plot (left) showing a significant positive correlation between mRNA expression levels of NQO1 and IC50 values of cisplatin across multiple bladder cancer cell lines. The 50% inhibiting concentration (IC50) dose‐response curves (right) for cisplatin treatment in T24, J82, and MB49 cells. (B) ZIP synergy score analysis using SynergyFinder reveals a strong additive effect between SFII and cisplatin in T24, J82, and MB49. (C,D) Flow cytometry‐based Annexin V‐EGFP/Propidium Iodide staining (C) showing dose‐dependent induction of apoptosis by SFII alone or in combination with cisplatin in T24, J82, and MB49 cells, *n* = 2 independent experiments. (D) Bar graphs summarizing apoptosis percentages at increasing concentrations of SFII in the presence of 0, 25, or 50 µm cisplatin. Synergistic enhancement of apoptosis is observed in both human and mouse cell lines. (E) Pharmacokinetic (PK) experiment was quantified using a validated LC–MS/MS method, showing the mean plasma concentration‐time curve over time after intraperitoneal administration of 60 mg/kg SFII to mice (*n* = 5 mice per time point, total *n* = 25 mice). The elimination half‐life (t_1/2_) was estimated to be 8.37 h, with an AUC_0–∞_ of 3,873.5 h·ng/mL, and plasma concentrations decreased to near the lower limit of quantification (∼1 ng/mL) by 24 h. (F) Schematic diagram of the in vivo study design, including subcutaneous and orthotopic bladder model. (G,J) Combination therapy study of SFII (60 mg/kg, i.p.) and cisplatin (3 mg/kg, i.p.) in subcutaneous bladder cancer model, *n* = 6 C57 male mice for each group. Representative images of excised tumors from each treatment group at endpoint (G). Tumor volume growth curves over 24 days (H). Tumor weights at endpoint confirm a dramatic reduction in the combination group (I). Body weight changes over time during treatment, no significant body weight loss was observed in any group (J). (K,L) Validation of combination therapy in an orthotopic bladder cancer model, *n* = 6 C57 male mice for each group. SFII inhibits the growth of bladder tumors, and the combination treatment (SFII + Cisplatin) significantly reduced bladder tumor volume (K) and weight (L) compared to monotherapies at endpoint. Correlations between continuous variables were determined using Spearman's correlation coefficient (A). Data are shown as mean ± SEM. The p‐values were calculated using one‐way (I and L) and two‐way (H) ANOVA, followed by Tukey's post hoc test. A *p*‐value < 0.05 is considered statistically significant. Statistical analysis was carried out using R (v4.2.1) or GraphPad Prism (v8.0.2).

As mentioned earlier, high‐risk patients with the T1HG1 subtype mostly show non‐responsiveness to BCG (Figure 1B,C). Although the guidelines recommend early cystectomy for these BCG‐refractory patients, there are still many patients who want to preserve the bladder. In light of cisplatin's status as a first‐line chemotherapy for UCB and its known induction of reactive oxygen species (ROS) leading to apoptotic cell death in T24 and J82 cells [43], we tested whether NQO1 inhibitors (particularly SFII, which has minimal side effects) could augment cisplatin's pro‐apoptotic effects in resistant tumors, supporting bladder‐preserving combination therapy. We first assessed the relationship between *NQO1* expression and cisplatin sensitivity. A strong positive correlation was observed between *NQO1* mRNA levels and cisplatin IC50 values (R = 0.54, *p* < 2.2e–16; Figure 5A), indicating that higher *NQO1* expression predicts greater chemoresistance. Additionally, we determined the IC50 values of three cell lines (Figure 5A), with J82 and T24 showing IC50 levels that correlated with their malignancy, consistent with a previous report [43].

Using the ZIP synergy algorithm (SynergyFinder [44]), which employs synergy scoring models to compare observed drug combination responses against expected responses, we evaluated drug interactions between SFII and cisplatin in vitro. The analysis revealed additive effects across all tested cell lines (T24 ZIP score: 1.695, J82 ZIP score: –5.17, and MB49 ZIP score: –3.786; Figure 5B; Figure S5A). Consistently, flow cytometry using Annexin V/PI staining demonstrated that SFII alone induced dose‐dependent apoptosis in all cell lines (Figure 5C,D). When combined with cisplatin (25 or 50 µm), apoptotic populations were significantly increased compared to either agent alone in all cell lines, confirming enhanced cell death through dual targeting (Figure 5C,D).

To better conduct in vivo drug experiments, we first characterized the pharmacokinetic (PK) profile of SFII to validate the rationale for the 48 h intermittent dosing regimen used in our subsequent in vivo efficacy studies. Following a single intraperitoneal dose of 60 mg/kg, SFII is rapidly absorbed (C_max_: 9839.345 ng/mL at 0.25 h) and cleared with an elimination half‐life (t_1/2_) of 8.373 h (Figure 5E; Tables S3 and S4). By 24 h, plasma concentrations approach the lower limit of quantification, confirming that the 48 h dosing interval prevents drug accumulation while ensuring sufficient systemic exposure. These PK properties established a clear pharmacological basis for the chosen regimen to support its translational potential.

To validate the antitumor efficacy in vivo, we established subcutaneous MB49 xenografts in C57 mice and treated them with vehicle, cisplatin, SFII, or their combination (Figure 5F). Consistent with representative images (Figure 5G), the combination group (SFII + cisplatin) showed the most pronounced tumor growth suppression, as evidenced by significantly reduced tumor volume over time (*p* < 0.0001 vs. all other groups; Figure 5H) and lower tumor weights at endpoint (*p* < 0.01 vs. vehicle; *p* < 0.05 vs. monotherapies; Figure 5I). As a complementary validation, we also tested dicoumarol, another classic NQO1 inhibitor in vivo studies (Figure S5B). Dicoumarol alone significantly suppressed tumor growth, meanwhile, its combination with cisplatin also significantly resulted in reduced tumor volume (*p* < 0.0001) and weight (*p* = 0.0073) compared to monotherapies (Figure S5C,D). Importantly, no significant body weight loss was observed in any group, indicating good systemic tolerability of the regimens, including the combination (Figure 5J; Figure S5E). Meanwhile, histopathology showed no severe kidney damage (Figure S5F), further supporting the safety and efficacy of NQO1 inhibition in combination with cisplatin. To further mimic clinical settings, we also established an orthotopic MB49 bladder cancer model (Figure 5F). The SFII alone significantly suppressed tumor growth (Figure 5K). Meanwhile, its combination with cisplatin demonstrated superior antitumor efficacy compared to monotherapies (combination vs. vehicle: *p* < 0.0001; combination vs. cisplatin: *p* = 0.0152; combination vs. SFII: *p* = 0.0074), consistent with the results of the subcutaneous model (Figure 5K,L).

Overall, our study results demonstrate that SFII can be metabolized normally in vivo as a drug and significantly enhances tumor cell sensitivity to cisplatin, thereby promoting apoptosis and inhibiting tumor growth with extremely low toxicity. This provides a combination therapy strategy with potential for bladder preservation for high‐risk T1HG1 subtype bladder cancer patients who are refractory to BCG treatment.

### 2.6 NQO1 Drives Immune Evasion by Reprogramming Macrophages to Limit T Cell Recruitment

**FIGURE 6:**
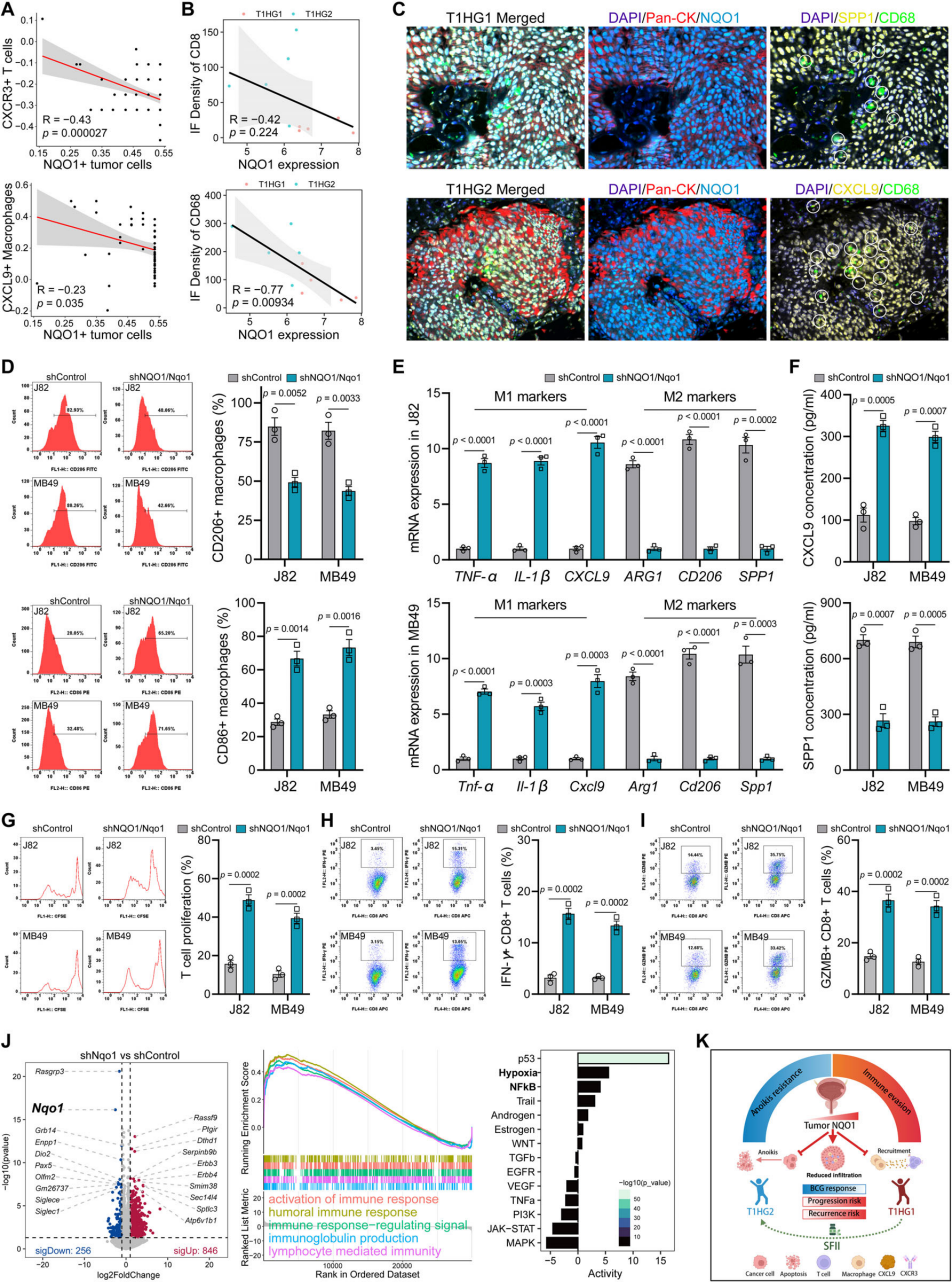
NQO1 drives immune evasion through modulation of immune infiltration and recruitment in bladder cancer. (A) Scatter plots showing negative correlations between *NQO1* expression in tumor cells and the signature fraction of CXCR3^+^ T cells (left) and CXCL9^+^ macrophages (right). (B) Correlation between NQO1 expression and immune cell density according to analysis of multiplex immunofluorescence (mIF) images. Higher NQO1 levels are associated with lower CD8^+^ T‐cell density and are inversely correlated with CD68^+^ macrophage density. (C) mIF imaging of representative T1HG1 and T1HG2 tumors, *n* = 3 patients per subtype. Top row (T1HG1): DAPI (blue), Pan‐CK (red), NQO1 (cyan); SPP1 (red), CD68 (green). Bottom row (T1HG2): DAPI (blue), Pan‐CK (red), NQO1 (green); CXCL9 (red), CD68 (green). Circles highlight co‐localization of CD68^+^ macrophages with CXCL9 or SPP1. (D) Flow cytometry analysis of macrophage polarization markers (CD86^+^ M1 vs. CD206^+^ M2) in macrophages co‐cultured with shControl or shNQO1 bladder cancer cells. (E) qRT‐PCR analysis of M1/M2 polarization markers and immune‐related genes in macrophages following co‐culture with tumor cells. (F) ELISA quantification of CXCL9 and SPP1 protein levels in the supernatants of co‐cultured macrophages. (G) CFSE dilution assay assessing the proliferation of CD8^+^ T cells after co‐culture with macrophages pre‐conditioned by shControl or shNQO1 tumor cells. (H,I) Flow cytometry analysis of intracellular IFN‐γ (H) and Granzyme B (I) expression in CD8^+^ T cells. (J) Volcano plot of differentially expressed genes in shNqo1 vs. shControl MB49 xenografts (left). GSEA enrichment plots showing activation of immune response pathways in NQO1‐knockdown tumors (middle). Pathway activity scores derived from DEGs in shNqo1 vs. shControl (right). (K) Conceptual model summarizing the dual role of tumor NQO1 in T1HG bladder cancer progression, created using BioRender (https://biorender.com). Correlations between continuous variables were determined using Spearman's correlation coefficient (A and B). Each data point represents an individual sample. Data are shown as mean ± SEM. The *p*‐values were calculated using two‐tailed unpaired Student's *t*‐test (D, E, F, G, H, and I). A *p*‐value < 0.05 is considered statistically significant. Statistical analysis was carried out using R (v4.2.1) or GraphPad Prism (v8.0.2).

Our aforementioned results indicate that immune microenvironment suppression is a key molecular characteristic of the T1HG1 subtype with high NQO1 expression. Therefore, we further sought to elucidate whether tumor cells with high NQO1 expression drive immune evasion by reprogramming the states of other immune cells. Analysis of bulk RNA‐seq data in the discovery cohort revealed a significant negative correlation between NQO1 expression and the signature fractions of CXCR3^+^ T cells and CXCL9^+^ macrophages (Figure 6A). This was further confirmed by mIF staining in patients from the external validation cohort (Figure 6B). Consistent with these findings, mIF staining analysis showed that T1HG1 tumors (high NQO1) exhibited prominent SPP1^+^ macrophage infiltration, whereas T1HG2 tumors (low NQO1) showed enriched CXCL9^+^ macrophages (Figure 6C).

To establish a causal link between tumor NQO1 and macrophage polarization, we employed a transwell co‐culture system. Human J82 or murine MB49 bladder cancer cells stably expressing shControl or shNQO1/shNqo1 were co‐cultured with THP‐1–derived or RAW264.7 macrophages, respectively. Flow cytometry analysis revealed that macrophages co‐cultured with shNQO1/shNqo1 tumor cells displayed a significantly increased M1/M2 ratio, characterized by elevated CD86 expression and reduced CD206 expression, compared to those co‐cultured with shControl cells (Figure 6D). Correspondingly, qRT‐PCR analysis confirmed that macrophages exposed to NQO1‐knockdown tumor cells exhibited upregulated expression of pro‐inflammatory M1‐related genes (*TNF‐α*, *IL‐1β*, *CXCL9*) and downregulated expression of M2‐associated markers (*ARG1*, *CD206*, *SPP1*) (Figure 6E). ELISA further validated that NQO1 knockdown in tumor cells significantly increased CXCL9 secretion and decreased SPP1 secretion by macrophages (Figure 6F).

We next investigated whether NQO1‐mediated macrophage reprogramming functionally impacts T cell responses. Macrophages pre‐conditioned by co‐culture with tumor cells were subsequently co‐cultured with CD8^+^ T cells. CFSE dilution assays demonstrated that CD8^+^ T cells co‐cultured with macrophages pre‐conditioned by shNQO1 tumor cells exhibited significantly enhanced proliferative capacity (Figure 6G). Furthermore, flow cytometry analysis revealed that these CD8^+^ T cells showed markedly increased expression of the effector molecules IFN‐γ (Figure 6H) and Granzyme B (Figure 6I), indicating restored cytotoxic potential.

To validate these findings in vivo, we performed transcriptomic profiling of shNqo1 and shControl MB49 xenografts. Volcano plots identified 846 upregulated and 256 downregulated genes (|log_2_FC| > 1, FDR < 0.05) in shNqo1 tumors (Figure 6J). Gene set enrichment analysis (GSEA) revealed significant enrichment of immune response and lymphocyte‐mediated immunity pathways, indicating that NQO1‐knockdown tumors reprogram the tumor immune microenvironment (Figure 6J). Consistently, CIBERSORTx deconvolution analysis demonstrated a pronounced shift in macrophage polarization, with a relative increase in M1 macrophages and a decrease in M2 macrophages in the NQO1‐knockdown group (Figure S6), corroborating the transition toward an immunostimulatory microenvironment.

Collectively, our findings establish a mechanistic link between NQO1‐mediated anoikis resistance and immune evasion in T1HG1‐subtype tumors (Figure 6K). Tumor‐derived NQO1 drives immune suppression by reprogramming macrophages toward an immunosuppressive M2‐like phenotype (characterized by high SPP1 and low CXCL9), which in turn inhibits CD8^+^ T cell recruitment and effector function. This creates a permissive niche for tumor progression and BCG resistance. Consequently, targeting NQO1 with inhibitors such as SFII represents a promising dual‐targeting strategy: it not only restores chemosensitivity and induces tumor apoptosis but also reactivates anti‐tumor immunity by reversing macrophage polarization. This dual action addresses both the intrinsic survival mechanisms and the extrinsic immunosuppressive microenvironment of T1HG1 tumors.

### 2.7 T1HG‐UCBguider Enables Robust Risk Stratification and Treatment Guidance across Cohorts

**FIGURE 7:**
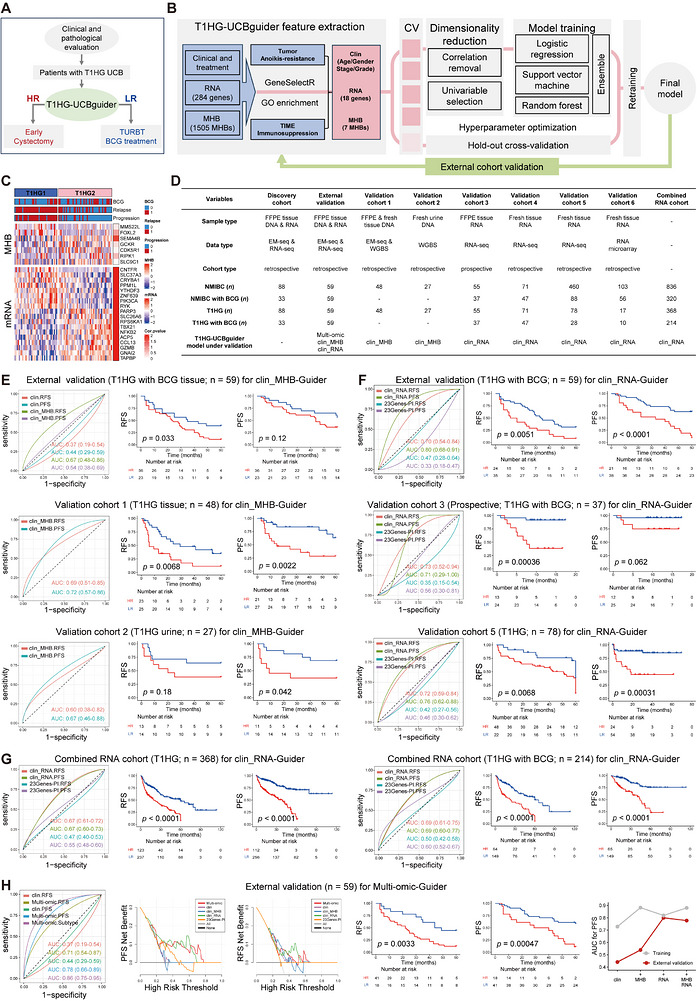
Development and multi‐cohort validation of the T1HG‐UCBguder model for risk stratification and treatment guidance in bladder cancer. (A) Potential clinical impact of the T1HG‐UCBguider for risk stratification and treatment selection in T1 high‐grade bladder cancer. (B) Schematic of the machine learning framework. (C) Heatmap displaying the association between the feature levels of markers and prognosis in two subtypes from discovery cohort. (D) A brief overview of the multi‐center cohorts (*n* = 8) included into the model development and validation phases. (E) Risk stratification performance of clin_MHB‐Guider with potential applicability to urine biopsy. AUC, recurrence‐free survival (RFS), and progression‐free survival (PFS) curves were generated to evaluate the performance of the model in predicting progression and recurrence from external validation cohort (*n* = 59; tissue specimens from BCG‐treated patients), validation cohort 1 (*n* = 48; tissue specimens), and validation cohort 2 (*n* = 27; urine samples). (F) Comparative evaluation of clin_RNA‐Guider vs. 23‐Gene Prognostic Index (23Genes‐PI) in T1HG UCB. AUC (left), RFS (middle), and PFS (right) curves were generated for performance benchmarking in risk stratification and identifying high‐risk patients with BCG‐refractory disease from external validation cohort (*n* = 59; T1HG BCG), the prospective validation cohort 3 (*n* = 37; T1HG BCG), and the validation cohort 5 (*n* = 37; T1HG BCG). (G) To fairly compare the performance of the two models, the combined RNA cohort included all available RNA data from all cohorts, including the training set for the 23‐Gene Prognostic Index. The performance benchmarking of two models was performed in the T1HG subset (*n* = 368) and the BCG‐treated T1HG subset (*n* = 214) of the combined RNA cohort. (H) External validation of the Multi‐omic‐Guider (*n* = 59). The integrated model demonstrates superior performance compared to single‐input models, with RFS.AUC = 0.71 and PFS.AUC = 0.78 (left). Net benefit analysis using decision curve analysis (DCA) shows higher clinical utility across various risk thresholds (middle). Kaplan‐Meier curves demonstrate worse RFS and PFS in high‐risk patients. AUCs with 95% CI were labeled in all ROC plots (E–H). The log‐rank test was used to compare the Kaplan–Meier survival curves (E–H). A *p*‐value < 0.05 is considered statistically significant. Statistical analysis was carried out using R (v4.2.1).

To address the critical challenge of inaccurate risk stratification and treatment selection in T1HG bladder cancer, these above findings motivated the development of a machine learning framework [45] to integrate the NQO1‐mediated dual features for predicting prognosis risk and BCG treatment response (Figure 7A). We screened and identified 18 genes involved in anoikis resistance or immune suppression pathways from 284 prognostic genes using GeneSelectR (Figure S7A–C). Additionally, 7 MHB markers were selected from 1505 candidates, showing an inverse correlation between methylomic and transcriptomic changes (Figure S7D,E). Using multi‐omic subtypes as binary classification labels, we integrated 18 mRNA features or 7 MHB features along with 4 clinical features (age, gender, stage, and grade) into three sub‐models (clin_RNA‐Guider, clin_MHB‐Guider, and Multi‐omic‐Guider) by the machine learning framework, collectively termed T1HG‐UCBguider (Figure 7B). These prognostic features showed significant differences between subtypes (Figure S7C,D) and were expressed in various cell types within the tumor microenvironment (Figure S7F), retaining the key subtype‐specific signatures (Figure 7C).

To validate the three sub‐models of T1HG‐UCBguider, seven independent validation cohorts and a combined RNA cohort containing samples of all available transcriptome data in this study were included in addition to the discovery cohort (Figure 7D, Table 1). Except for the validation cohort 3 as a prospective cohort, all other cohorts were retrospective. The external validation cohort comprised paired whole‐genome DNA methylation and transcriptomic data, whereas validation cohorts 1 and 2 exclusively contained DNA methylation data. Validation cohorts 3–6 solely included transcriptomic data.

**TABLE 1:** Clinical characteristics of the eight cohorts used to develop and validate the T1HG‐UCBguider models.

Variables	No. (%)							
	Discovery cohort (n = 88)	External cohort (n = 59)	Validation cohort 1 (n = 48)	Validation cohort 2 (n = 27)	Validation cohort 3 (n = 55)	Validation cohort 4 (n = 71)	Validation cohort 5 (n = 460)	Validation cohort 6 (n = 103)
Gender								
Male	74 (84)	51 (86)	34 (71)	21 (78)	38 (69)	NA	357 (78)	87 (84)
Female	14 (16)	8 (14)	14 (29)	6 (22)	17 (31)	NA	103 (22)	16 (16)
Age, median (range), y	69 (39–88)	66 (32‐86)	66.5 (46–88)	66 (47‐87)	70 (37‐91)	NA	69 (24‐96)	66 (24‐88)
T stage								
CIS	0 (0)	0 (0)	0 (0)	0 (0)	0 (0)	0 (0)	3 (< 1)	0 (0)
Ta	0 (0)	0 (0)	0 (0)	0 (0)	0 (0)	0 (0)	345 (75)	24 (23)
T1	88 (100)	59 (100)	48 (100)	27 (100)	55 (100)	71 (100)	112 (24)	79 (77)
Grade								
High‐grade	88 (100)	59 (100)	48 (100)	27 (100)	55 (100)	71 (100)	176 (38)	17 (17)
Low‐grade	0 (0)	0 (0)	0 (0)	0 (0)	0 (0)	0 (0)	277 (60)	86 (83)
PUNLMP	0 (0)	0 (0)	0 (0)	0 (0)	0 (0)	0 (0)	7 (2)	0 (0)
BCG treatment								
Yes	33 (38)	59 (100)	12 (25)	18 (67)	37 (67)	47 (66)	88 (19)	56 (54)
No	55 (62)	0 (0)	36 (75)	9 (33)	18 (33)	23 (32)	372 (71)	47 (46)
Unknown	0 (0)	0 (0)	0 (0)	0 (0)	0 (0)	1 (2)	0 (0)	0 (0)
Progression								
Yes	42 (48)	33 (56)	21 (44)	12 (44)	8 (15)	9 (13)	31 (7)	14 (14)
No	46 (52)	26 (44)	27 (56)	15 (56)	47 (85)	62 (87)	429 (93)	89 (86)
Recurrence								
Yes	56 (64)	46 (78)	33 (69)	13 (48)	16 (29)	29 (41)	264 (57)	36 (35)
No	32 (36)	13 (22)	15 (31)	14 (52)	39 (71)	42 (59)	150 (33)	67 (65)
Unknown	0 (0)	0 (0)	0 (0)	0 (0)	0 (0)	0 (0)	46 (10)	0 (0)
Median follow‐up (months)	44.95	51	29.13	60	11.3	36	36	89.5
Enrollment timeline	2009‐2021	2016‐2020	2017‐2021	2015‐2018	2022‐2023	NA	NA	NA

Abbreviations: BCG, Bacillus Calmette‐Guérin; CIS, Carcinoma in situ; NA, not available, PUNLMP, papillary urothelial neoplasm of low malignant potential.

In BCG‐treated patients, the clin_MHB‐Guider stratified individuals into high‐risk (HR) and low‐risk (LR) groups with different progression and recurrence rates (log‐rank, *p =* 0.033) in the external validation cohort (Figure 7E). This performance is confirmed in validation cohort 1, where the model achieves robust predictive accuracy (RFS‐AUC = 0.69, 95% CI: 0.51–0.85; PFS‐AUC = 0.72, 95% CI: 0.57–0.86), and HR patients exhibit higher recurrence (log‐rank, *p =* 0.0068) and progression (log‐rank, *p =* 0.0022) (Figure 7E). Consistent with our previous findings of elevated tumor‐derived signals in urine from UCB patients that enable non‐invasive diagnosis and recurrence monitoring [46, 47], the model retained predictive value in preoperative urine samples from 27 T1HG UCB patients, supporting non‐invasive risk assessment (RFS‐AUC = 0.60, 95% CI: 0.38–0.82; PFS‐AUC = 0.67, 95% CI: 0.46–0.88; log‐rank, *p =* 0.042; Figure 7E).

Recently, a 23‐Gene Prognostic Index (23Genes‐PI) can identify patients with high‐risk NMIBC with distinct progression and BCG treatment responses [14]. Benchmarking against the 23Genes‐PI model demonstrates that the clin_RNA‐Guider achieves superior performance for risk stratification and BCG response prediction across multiple cohorts, including the external validation cohort (PFS‐AUC: 0.80 vs. 0.33; RFS‐AUC: 0.70 vs. 0.47), the prospective cohort (PFS‐AUC: 0.71 vs. 0.56; RFS‐AUC: 0.73 vs. 0.35), and the combined RNA cohort (PFS‐AUC: 0.69 vs. 0.60; RFS‐AUC: 0.69 vs. 0.50) (Figure 7F,G; Figure S8). This advantage is consistently observed across multi‐center T1HG cohorts and extends to NMIBC populations, with improved identification of BCG‐refractory high‐risk patients, particularly for progression (Figure S8). Collectively, these results support the robustness, scalability, and clinical utility of the clin_RNA‐Guider.

The Multi‐omic‐Guider stratified patients into high‐ and low‐risk groups with significantly different progression and recurrence rates in both the discovery cohort and its BCG‐treated subset (Figure S7G,H). Its predictive value for intravesical BCG response is further validated in the external validation cohort, outperforming clinical parameters in predicting recurrence (AUC = 0.71; 95% CI, 0.54–0.87) and progression (AUC = 0.78; 95% CI, 0.66–0.89) (Figure 7H). Importantly, this model demonstrates net clinical benefit and identifies high‐risk patients with worse RFS (*p =* 0.0033) and PFS (*p =* 0.00047) after BCG treatment, supporting early cystectomy in BCG‐refractory cases (Figure 7H). Notably, similar to the reported multi‐omic model [45], although increasing omics layers did not always improve AUC, the integrated model shows more stable performance (Figure 7H).

Importantly, ethnicity‐stratified analyses further confirmed the robustness of the model across populations. As shown in Figure S9, consistent risk stratification and comparable predictive performance were observed in both Caucasian and Asian cohorts, with no significant interaction (*p* > 0.05) between ethnicity and risk score. Additionally, the calibration plots for all validation cohorts demonstrated that model predictions are well calibrated across diverse cohorts and detection platforms (Figure S10). These findings support the cross‐population applicability of the T1HG‐UCBguider.

Overall, our approach integrates clinical variables with mRNA‐based and/or DNA methylation‐based biomarkers into a machine learning framework to enable individualized risk assessment and treatment selection. To enhance transparency and accessibility, we developed a user‐friendly web interface for T1HG‐UCBguider (Figure S11), now publicly available at https://t1hg‐ucbguider.site/omics‐guider.

## 3 Discussion

T1 high‐grade bladder cancer remains one of the most clinically challenging forms of non–muscle‐invasive disease. Current risk stratification relies heavily on histopathology, which fails to capture the underlying biological heterogeneity and often leads to both overtreatment and delayed intervention. In this study, we establish a multi‐omic framework that defines a clinically actionable molecular subtype, T1HG1, which is characterized by aggressive behavior, resistance to Bacillus Calmette–Guérin (BCG) therapy, and a distinct immune‐suppressed state.

By integrating whole‐genome methylation and transcriptomic data from FFPE tissues, we generated a high‐quality dataset for T1HG bladder cancer and identified a coherent molecular entity rather than a purely statistical subtype. T1HG1 is defined by coordinated genomic instability, epigenetic reprogramming, and transcriptional remodeling. Importantly, this subtype shows consistent prognostic value across independent cohorts and outperforms conventional clinicopathological variables in predicting both recurrence and progression.

From a clinical perspective, T1HG1 identifies a group of patients with markedly elevated risk. These patients show substantially higher progression rates and are more likely to require radical cystectomy within five years. These findings highlight the limitations of current guideline‐based management and support the need for early molecular stratification to guide treatment decisions [7].

Mechanistically, our data reveal that the aggressive phenotype of T1HG1 is driven by two interconnected biological programs: resistance to anoikis and suppression of antitumor immunity. Anoikis resistance enables tumor cells to survive under detachment stress and supports invasive behavior through metabolic adaptation and redox regulation [31, 33, 48, 49]. At the same time, T1HG1 tumors exhibit an immune‐cold microenvironment, with reduced infiltration of CD8^+^ T cells and M1‐like macrophages, enrichment of exhausted PD‐1^+^ T cells, and impaired CXCL9–CXCR3 signaling. In contrast, T1HG2 tumors retain immune activity and show features consistent with responsiveness to BCG [31, 32, 50, 51, 52, 53]. These observations provide a biological explanation for treatment heterogeneity in T1HG disease.

A central finding of this study is the identification of NQO1 as a key regulator linking these two programs. NQO1 is consistently upregulated in T1HG1 across bulk, single‐cell, and protein‐level analyses and is associated with poor clinical outcomes. Functional experiments demonstrate that inhibition of NQO1 restores sensitivity to anoikis and enhances cisplatin‐induced apoptosis. Both genetic knockdown and pharmacological inhibition using skullcapflavone II and dicoumarol produce consistent effects. Notably, skullcapflavone II shows favorable tolerability in vivo and avoids the safety limitations associated with classical inhibitors such as dicoumarol [41]. Pharmacokinetic data support adequate exposure and efficient clearance, consistent with effective activity and safe metabolism in vivo.

Beyond tumor‐intrinsic effects, NQO1 also can regulate the tumor immune microenvironment [54, 55, 56, 57, 58]. Consistent with previous findings that NQO1 promotes M2‐like macrophage polarization and contributes to an immunosuppressive niche in hepatocellular carcinoma [54, 58], our data extend this concept by establishing a direct causal link between tumor‐derived NQO1 and macrophage reprogramming in bladder cancer. Using transwell co‐culture systems, we demonstrate that tumor NQO1 acts as a master regulator, instructing macrophages to adopt an immunosuppressive M2‐like phenotype characterized by high SPP1 and low CXCL9 expression. Mechanistically, this NQO1‐mediated reprogramming suppresses CXCL9 secretion, thereby impairing the recruitment of CXCR3^+^ T cells and creating a “cold” tumor microenvironment. Consequently, the resulting impairment in CD8^+^ T cell proliferation and effector function further facilitates immune evasion. This crosstalk highlights NQO1 not merely as a metabolic enzyme, but as a central node coordinating the cross‐talk between tumor survival signals and immune suppression. Although direct transcriptional regulation of CXCL9 by NQO1 requires further validation, the consistent changes observed across co‐culture systems, ELISA, and in vivo transcriptomic profiling strongly support a functional link between tumor‐derived NQO1 and macrophage CXCL9 suppression. (e.g., ATAC‐seq, ChIP‐seq, or FOODIE [59]) will be necessary to elucidate the precise transcriptional or epigenetic mechanisms by which NQO1 signaling modulates macrophage gene expression, such as the regulation of CXCL9 and SPP1 loci.

The dual role of NQO1 in promoting tumor survival and suppressing immunity provides a strong rationale for therapeutic targeting. Inhibition of NQO1 disrupts both anoikis resistance and immune evasion, which creates a therapeutic vulnerability. In preclinical models, skullcapflavone II enhances the efficacy of cisplatin and induces robust tumor suppression with minimal toxicity. These findings support a combination strategy aimed at bladder preservation in patients who are refractory to BCG.

To facilitate clinical translation, we developed T1HG‐UCBguider, a machine learning framework that integrates clinical variables with transcriptomic and methylation features associated with anoikis resistance and immune suppression. The model shows consistent performance across seven independent cohorts, including urine‐based methylation datasets. It outperforms the 23‐Gene Prognostic Index in predicting progression and BCG failure in T1HG UCB. These results indicate that molecularly informed models can improve risk stratification and guide treatment selection in clinical practice.

Several limitations should be considered. First, although multiple cohorts were included, the prospective validation cohort remains relatively small. Larger prospective studies are needed to confirm clinical utility. Second, the study population is enriched for Asian patients, which may introduce population bias. Validation in more diverse cohorts will be important to assess generalizability. Third, the current framework does not incorporate proteomic or spatial omics data. As a result, immune interactions are inferred primarily from bulk and limited spatial analyses. Future integration of spatial transcriptomics and imaging‐based proteomics will provide more detailed insights into tumor–immune architecture. Finally, although skullcapflavone II shows promising preclinical activity, its pharmacokinetics, optimal dosing, and long‐term safety in humans remain to be established. In addition, the interaction between NQO1 inhibition and BCG‐induced immune responses warrants further investigation.

In summary, this study defines a high‐risk molecular subtype of T1HG bladder cancer driven by coordinated anoikis resistance and immune evasion. NQO1 acts as a central regulator of this phenotype and represents a promising therapeutic target. By integrating mechanistic insights with predictive modeling, our work provides a framework for precision management and supports the development of bladder‐preserving strategies in high‐risk patients.

## 4 Methods

### 4.1 Patients and Samples

This multi‐center, ambispective cohort study was approved by the Ethics Committee of Peking University First Hospital (ethical number: 2022SR338). All methods were performed in accordance with relevant guidelines and regulations. Informed consent was obtained from each participant or their immediate families. The study utilized clinical, methylomic, and/or transcriptomic data from 928 UCB patients across eight cohorts. Sequencing data for model development and validation were obtained from 5 participating cohorts in China, 1 in Korea, and 2 from the European Union (EU). Discovery cohort (*n* = 88), external validation cohort (*n* = 59), and validation cohort 1 (*n* = 48) were retrospectively collected, and validation cohort 3 (*n* = 55) was prospectively registered (Registration number: ChiCTR2200064248). The main outcomes were progression‐free survival and recurrence‐free survival during a 5‐year follow‐up. Detailed baseline patient characteristics are outlined in Table 1. Additionally, two single‐cell RNA‐seq (scRNA‐seq) datasets of T1HG UCB were included [60].

### 4.2 Construction of Clinically Relevant Multi‐Omic Classification

In the discovery cohort of 88 patients, we conducted single‐omics and multi‐omic subtype analyses for prognostic evaluation. Initially, univariate Cox regression identified features associated with progression‐free survival (PFS) in the entire cohort and a subset of 33 patients treated with BCG treatment (*p* ≤ 0.05). Overlapping features across single‐omics data types (CNV, promoter methylation, MHBs, and gene expression) were selected for subtype classification. These included 607 CNV‐associated genes, 156 promoters, 1505 MHBs, and 284 mRNA features. CNV and methylation data were preprocessed by retaining features (gene‐level or 10‑kb bin‐level) with non‑missing values in at least 90% of samples; features with more than 10% missing values across samples were excluded to ensure robustness of subsequent multi‑omic analyses. Average methylation levels (AML) were calculated for each gene's promoter region (2000 bp upstream and 200 bp downstream of the transcription start site), and AML and MHL were computed for each MHB. MHBs present in >10% of samples were discarded, and missing values were imputed using the median for each MHB. Additionally, log2‐transformed TPM values, GSVA scores, and ssGSEA scores were calculated for gene expression, with genes having NAs in >10% of samples being filtered out and remaining NAs replaced by the median value. Single‐omic subtype classification was then performed using the “Execute.CC” function from CancerSubtypes (v1.24.0) [61], and multi‐omic subtype classification was conducted using the “ExecuteSNF.CC” function. Survival analysis was performed for both single‐omics and multi‐omic subtypes, generating survival curves for recurrence‐free survival (RFS), PFS, metastasis‐free survival, and overall survival within a five‐year timeframe. Multi‐factor Cox regression analysis based on PFS and RFS was conducted for the multi‐omic subtype to assess the impact of multiple factors on patient outcomes.

### 4.3 Immunohistochemistry and Immunofluorescence Staining Assays

FFPE tissue samples were sectioned into 5 µm slices and mounted on glass slides preheated to 70°C for 1 h at Peking University First Hospital. The slides were deparaffinized in xylene and rehydrated through graded ethanol solutions (100%, 95%, and 70%). Slides were then used for hematoxylin and eosin (H&E), terminal deoxynucleotidyl transferase dUTP nick‐end labeling (TUNEL), periodic acid‐Schiff (PAS), immunohistochemistry (IHC), or immunofluorescence (IF) staining. For H&E staining, sections were stained with hematoxylin for nuclear visualization and counterstained with eosin for cytoplasmic detail, followed by dehydration and mounting. The TUNEL staining was performed using a DAB (SA‐HRP) Tunel Cell Apoptosis Detection Kit (Servicebio; Cat. no. G1507‐50T) according to the manufacturer's instructions. For PAS staining, sections were oxidized in 0.5% periodic acid for 10 min, rinsed, and incubated in Schiff's reagent for 15 min to detect glycogen and glycoproteins, followed by hematoxylin counterstaining, dehydration, and mounting. For IHC or IF, slides were first treated to block endogenous peroxidase activity for 10 min, followed by antigen retrieval using citric acid solution using microwave heating for 15 min. After pre‐incubation with blocking buffer at room temperature for 10 min, slides were incubated overnight at 4°C with primary antibodies (detailed antibody information see Table S2). For IHC staining, the secondary antibody (HRP polymer, anti‐mouse/rabbit IgG) was incubated at room temperature for 20 min, followed by DAB staining, counterstained with hematoxylin, and imaged by microscopy. For IF staining, fluorescently conjugated secondary antibodies (anti‐mouse/rabbit IgG) were applied at 4°C for 30 min, followed by nuclear staining with DAPI. Multispectral images were captured using the 3DHISTECH PANNORAMIC system.

### 4.4 Cell Drug Experiments

Based on public RNA‐seq datasets of bladder cancer cell lines, the subtypes of J82 and T24 were identified using classifyT1HG, referencing classifyNMIBC [23, 24], based on differentially expressed genes (*p* < 0.01 and |logFC| > 1) from multi‐omic subtypes. The mRNA expression of NQO1 was measured by quantitative real‐time PCR (qRT‐PCR) using 5× FastKing‐RT SuperMix (TIANGEN, Cat. no. KR118‐02) and Taq Pro Universal SYBR qPCR Master Mix (Vazyme, Cat. no. Q712‐02). Cells were cultured at 37°C in 5% CO2. J82 and MB49 were cultured in DMEM medium (HyClone, Cat. no. SH30243.01), while T24 cells were cultured in RPMI 1640 medium (Gibco, Cat. no. 11835030), both supplemented with 1% penicillin/streptomycin antibiotic cocktail and 10% fetal bovine serum (VivaCell, Cat. no. C04001‐050X10). After treatment with different concentrations of skullcapflavone II (SFII) (Bestech, Cat. no. CFN92216), dicoumarol (Selleck, Cat. no. S4299), or cisplatin (MedChemExpress, Cat. no. HY‐17394), cells underwent the following assays. The 50% inhibiting concentration (IC50) of different drugs and combination response data for SFII and cisplatin at multiple doses for SynergyFinder [44] analysis were obtained using the Cell Counting Kit‐8 (ANALYSIS QUIZ, Cat. no. AQ308). Cell apoptosis was detected using the Annexin V‐EGFP/PI Cell Apoptosis Detection Kit (Servicebio, Cat. no. G1510) and flow cytometer (BD FACSAria II). Anoikis was simulated and detected using the CytoSelect 96‐well Anoikis Assay (CELL BIOLABS, Cat. no. CBA‐081), and cell viability was assessed by Multiskan FC 96‐well Plate Microplate Photometers.

### 4.5 NQO1/Nqo1 Knockdown of Bladder Cancer Cell Lines

Both murine (MB49) and human (J82) bladder cancer cell lines were transduced with lentiviral particles encoding short hairpin RNAs (shRNAs) targeting Nqo1/NQO1 or a non‐targeting control (shControl). Lentiviruses were generated by co‐transfecting HEK293T cells using a lentiviral packaging kit (Yeasen, Cat. no. 41102ES10), following the manufacturer's instructions. Viral supernatants were collected at 48 and 72 h post‐transfection, filtered through a 0.45‐µm membrane, and used to infect target cells in the presence of 8 µg/mL polybrene. After infection, stable knockdown cell lines were selected using puromycin (2–4 µg/mL) for 5–7 days. Knockdown efficiency was confirmed by qRT‐PCR. The resulting knockdown and control cell lines (J82 and MB49) were used for subsequent functional assays.

### 4.6 Cell Co‐Culture Experiments

Human bladder cancer J82 cells and murine MB49 cells with stable NQO1/Nqo1 knockdown (shNQO1‐J82, shNqo1‐MB49) or control (shControl‐J82, shControl‐MB49) were co‐cultured with THP‐1–derived macrophages or RAW264.7 macrophages, respectively. THP‐1 monocytes were differentiated into macrophages using 100 ng/mL PMA (MedChemExpress, Cat. no. HY‐18739) for 24 h. Co‐culture was performed using Transwell inserts with a 0.4 µm pore size (Corning, Cat. no. 3413). For human co‐culture, 1 × 10^5^ shControl‐J82 or shNQO1‐J82 cells were seeded in the lower chamber, and 1 × 10^5^ THP‐1–derived macrophages were seeded in the upper chamber. For murine co‐culture, 1 × 10^5^ shControl‐MB49 or shNqo1‐MB49 cells were seeded in the lower chamber, and 1 × 10^5^ RAW264.7 macrophages were seeded in the upper chamber. After 48 h of co‐culture, macrophages were harvested for phenotypic and molecular analyses.

Macrophage polarization was assessed by flow cytometry using CD86 PE (Santa Cruz Biotechnology, Cat. no. sc‐19617 PE) as an M1 marker and CD206 FITC (Santa Cruz Biotechnology, Cat. no. sc‐58987 FITC) as an M2 marker. Gene expression was quantified by qRT‐PCR. Total RNA was extracted using the RNAprep Pure Cell/Bacteria Kit (TIANGEN, Cat. no. DP430), and reverse transcription was performed using the PrimeScript RT Reagent Kit with gDNA Eraser (TaKaRa, Cat. no. RR047A). qPCR was conducted using TB Green Premix Ex Taq II (TaKaRa, Cat. no. RR820A) with specific primers for M2‐associated genes (*ARG1*/*Arg1*, *CD206*/*Cd206*, *SPP1*/*Spp1*) and pro‐inflammatory/chemokine genes (*TNF‐α*/*Tnf‐α*, *IL‐1β*/*Il‐1β*, *CXCL9*/*Cxcl9*), with *GAPDH* as reference gene. Protein levels of CXCL9 and SPP1 in culture supernatants were measured by ELISA using the Human/Mouse CXCL9 and SPP1 ELISA Kits (Jianglai Bio, Cat. no. JL14160 for human CXCL9, Cat. no. JL20226 for mouse Cxcl9; Cat. no. JL10368 for human SPP1, Cat. no. JL10068 for mouse Spp1).

To assess the functional consequences on adaptive immunity, macrophages pre‐conditioned by co‐culture with tumor cells (shControl, or shNQO1/shNqo1) were subsequently co‐cultured with CD8^+^ T cells. Human CD8^+^ T cells were isolated from PBMCs using the Human CD8 T Cell Isolation Kit (Thermo Fisher, Cat. no. 8804‐6812). Murine CD8^+^ T cells were isolated from mouse splenocytes using the Mouse CD8 T Cell Isolation Kit (Thermo Fisher, Cat. no. 8804‐6822). The pre‐conditioned macrophages (1 × 10^5^) were co‐cultured with CD8^+^ T cells (5 × 10^5^) at a ratio of 1:5 in 6‐well plates for 72 h. T cell proliferation was assessed by CFSE dilution using the CFSE Cell Division Tracker Kit (BioLegend, Cat. no. 423801). Activation markers, including IFN‐γ and Granzyme B, were measured by flow cytometry using IFN‐γ PE (BioLegend, Cat. no. 383403 for human, Cat. no. 505807 for mouse) and Granzyme B PE (BioLegend, Cat. no. 372208), following fixation and permeabilization with the Intracellular Staining Permeabilization Wash Buffer (10X) (BioLegend, Cat. no. 421002).

### 4.7 Animal Experiments

All protocols for mice were approved by the Animal Ethics Committee of Beijing Experimental Animal Research Center (ethic approval number: BLARC‐LAWER‐202502002 and BLARC‐LAWER‐202504011). Wild‐type (WT) C57 male mice (6–8 weeks) purchased from Speifu (Beijing) Biotechnology Co., Ltd. (Beijing, China) were housed in a pathogen‐free facility at 22°C with 50% humidity and a 12 h light and 12 h dark cycle.

Pharmacokinetic analysis of skullcapflavone II was performed in an independent cohort of mice to support the dosing strategy used in vivo. SFII was administered via intraperitoneal injection at 60 mg/kg (consistent with the therapeutic experiments). Blood samples were collected at pre‐dose and 0.25, 0.5, 1, 2, 4, 8, 12, 24, and 48 h after administration (*n* = 5 per time point; total N = 25). Plasma concentrations of SFII were quantified using a validated LC‐MS/MS method. Pharmacokinetic parameters, including C_max_, T_max_, t_1/2_, and AUC, were calculated using a non‐compartmental model.

For subcutaneous syngeneic tumor models, two sets of experiments were conducted. First, to evaluate the role of NQO1, MB49 cells stably transfected with shControl or NQO1‐knockdown (shRNA) plasmids were injected subcutaneously into mice (1 x 10^5^ cells in 100 µl PBS per mouse, *n* = 5 per group). Tumor volume was measured twice per week, and the experiment lasted for 30 days, after which tumor tissue was collected for immunostaining and RNA sequencing. Second, to assess therapeutic efficacy, MB49 cells (1 x 10^6^ cells in 100 µL PBS per mouse) were injected subcutaneously. Once the tumors reached 80–100 mm^3^, mice were randomly allocated into six groups (*n* = 6 per group) with similar average body weights, and then treated for 24 days with vehicle, cisplatin (3 mg/kg, 6 times, i.p.), dicoumarol (50 mg/kg, 12 times, i.p.), SFII (60 mg/kg, 12 times, i.p.), combination of dicoumarol + cisplatin, combination of SFII + cisplatin. The tumor volume of mice was measured three times weekly. At the end of the experiment, tumor tissues and kidney tissues were obtained for immunostaining or single cell RNA sequencing. Tumor volume = (a × a × b)/2 (a, the smallest diameter; b, the largest diameter).

To further mimic clinical settings, we also established an orthotopic bladder cancer model. This model was constructed based on a previous study [62]. In short, the mouse bladder mucosa was cauterized by a platinum wire electrode using a monopolar coagulation at 2 W for 1 s, and then MB49 cells (5x10^5^ in 100 µl PBS per mouse) were instilled into the bladder for 2 h. 12 days after tumor implantation, mice were randomized into four groups (n = 6 per group) and treated intraperitoneally for 12 days with vehicle, cisplatin, SFII, or cisplatin + SFII (same doses for the subcutaneous model). Finally, bladder tumors were collected for weight measurement.

### 4.8 T1HG‐UCBguider Construction and Validation

GeneSelectR (v1.0.1) (https://github.com/dzhakparov/GeneSelectR) and Gene Ontology (GO) enrichment were used to refine features from the initial pool of 1505 Methylation Haplotype Blocks (MHBs) and 284 genes used for multi‐omic subtype classification. Ultimately, 18 gene expression markers and 7 MHB markers were identified as biologically interpretable features associated with suppressive immune microenvironment and tumor anoikis resistance. Machine learning model construction followed previously described methods [45]. The 18 mRNA markers and 7 MHB markers were integrated with 4 clinical variables (age, gender, tumor stage, and grade) to develop two machine learning sub‐models within the T1HG‐UCBguider framework. These sub‐models, named “clin_RNA” and “clin_MHB”, were created using an ensemble of logistic regression, support vector machine, and random forest algorithms. T1HG‐UCBguider was designed to predict tumor recurrence, progression, and response to BCG treatment, and both sub‐models underwent validation across multiple cohorts. Additionally, the performance of the “clin_RNA” sub‐model was compared with the existing 23‐Gene Prognostic Index model [14] in a distinct cohort of T1HG UCB or NMIBC patients. This comparison aimed to assess the predictive accuracy and robustness of the “clin_RNA” sub‐model in a different clinical context. To ensure statistical rigor, model development employed a nested cross‐validation strategy with hyperparameter optimization, and feature selection was stabilized within the GeneSelectR framework. Internal validation of model discriminative ability was performed using bootstrapping to derive 95% confidence intervals for AUC values (via the pROC package with 2000 resamples). Model performance was assessed by the AUC‐ROC, Kaplan–Meier curves, and decision curve analysis. Detailed analysis methodologies are provided in the Supplementary Material. Finally, to enhance model transparency and accessibility, T1HG‐UCBguider was deployed on a user‐friendly web interface (https://t1hg‐ucbguider.site/omics‐guider).

### 4.9 Statistical Analysis

All statistical analyses were performed using R (v4.2.1) and GraphPad Prism (v8.0.2). Continuous variables are presented as mean ± SEM unless otherwise indicated. Data normality was assessed using the Shapiro–Wilk test prior to parametric testing. For comparisons between two groups, a two‐sided Student's *t*‐test or a Wilcoxon rank‐sum test was applied as appropriate. For multiple group comparisons, one‐way ANOVA followed by post hoc Tukey test or Kruskal–Wallis test was used. Survival analyses were performed using the Kaplan–Meier method with the log‐rank test. Cox proportional hazards models were used for multivariable analysis. Sample size (n) for each experiment is indicated in the corresponding figure legends. No statistical methods were used to predetermine sample size. *P* values < 0.05 were considered statistically significant unless otherwise specified. Where applicable, multiple testing correction was performed using the Benjamini–Hochberg method. All tests were two‐sided.

## Author Contributions

Conceptualization was contributed by Weimin Ci, Hongzhao Li, Xuesong Li, Yuan Liang, and Bin Guo. Funding acquisition was carried out by Weimin Ci, Xuesong Li, Yuan Liang, and Yue Shi. Patient enrollment, sample collection, and clinical information acquisition were performed by Chunru Xu, Shufan Fu, Jian Fan, and Changwei Yuan. Bioinformatic analysis and experiments were performed by Bin Guo, with the assistance of Qiang Cheng, Yuan Liang, and Juan Li. Project administration was performed by Chunru Xu, Mei Zhang, Qiang Cheng, Yanqing Gong, and Yue Shi. Supervision was provided by Weimin Ci, Hongzhao Li, Xuesong Li, and Yuan Liang. Technical and material support were provided by Linkuo Shang, Gaojie Li, Yang Yang, Ying Wang, Shengwei Xiong, Yifan Zuo, and Elena Papaleo. The original draft was written by Bin Guo, and review and editing were conducted by Bin Guo, Weimin Ci, Qiang Cheng, and Yuan Liang, with contributions from all authors.

## Funding

This work was supported by the National Key R&D Program of China (2023YFC2507002 to Yuan Liang and Yue Shi, 2023YFC3402704 to Yue Shi), the National High Level Hospital Clinical Research Funding (High Quality Clinical Research Project of Peking University First Hospital, 2022CR72 to Xuesong Li), the National Natural Science Foundation of China (82341030 and 82173061 to Weimin Ci, 82273350 to Xuesong Li, U23A20460 to Xuesong Li and Yuan Liang, 82103426 to Yuan Liang).

## Conflicts of Interest

The authors declare no conflicts of interest.

## Supporting information




**Supporting File**: advs75148‐sup‐0001‐SuppMat.docx.

## Data Availability

The original raw sequence data of our discovery cohort, external validation cohort, validation cohort 1, and validation cohort 3 reported in this paper have been deposited in the Genome Sequence Archive [63] in the National Genomics Data Center [64], China National Center for Bioinformation / Beijing Institute of Genomics, Chinese Academy of Sciences (GSA‐Human: HRA007427) that are publicly accessible at https://ngdc.cncb.ac.cn/gsa‐human. The public gene expression datasets for the validation cohort 4 [65], validation cohort 6 [66, 67] are available in the NCBI GEO public database (https://www.ncbi.nlm.nih.gov/geo/) under data series accession numbers GSE154261 and GSE13507. The public gene expression dataset for validation cohort 5 [22] is available in the ArrayExpress database (https://www.ebi.ac.uk/ arrayexpress/) under accession number E‐MTAB‐4321. The T1HG‐UCBguider was deployed in a user‐friendly web interface (https://t1hg‐ucbguider.site/omics‐guider), and its source code is available at GitHub (https://github.com/guobin8216/T1HG‐UCBguider). All other data reported in this paper is available from the lead contact upon request.
