# Supplementary material for: NQO1‐Mediated Anoikis Resistance and Immune Evasion Define a High‐Risk Multi‐Omic Subtype for Precision Management of T1 High‐Grade Bladder Cancer
Source: Adv Sci (Weinh). 2026 Apr 7;13(36):e23605. doi: 10.1002/advs.202523605 (PMC13317658; doi:10.1002/advs.202523605)
Supplement: Supplementary file 1 — Supporting File: advs75148‐sup‐0001‐SuppMat.docx. [file ADVS-13-e23605-s001.docx]

**Supplementary materials**

**Supplementary methods**

**DNA and RNA Extraction**

In the discovery cohort and external validation cohort, total nucleic acids (RNA and DNA) were extracted from 2–4 formalin-fixed and paraffin-embedded (FFPE) sections using the TruXTRAC® FFPE Total NA Kit (Covaris, Cat. no. PN 520220). RNA was quantified using a NanoDrop 2000 (Thermo Scientific), and DNA was quantified using a Qubit 4 Fluorometer (Invitrogen). For validation cohort 1, FFPE DNA was extracted with the Quick-DNA™ FFPE MiniPrep Kit (Zymo, Cat. no. R1009) and fresh tissue DNA with the QIAamp DNA Mini Kit (QIAGEN, Cat. no. 51304), followed by quantification using the Qubit 4 Fluorometer. In validation cohort 3, RNA was extracted using the Quick-RNA™ FFPE MiniPrep Kit (Zymo, Cat. no. R1008) and quantified with a Nanodrop 2000.

**Enzymatic Methylation-sequencing**

A mixture comprising genomic DNA (200 ng) and unmethylated λDNA (0.6 ng) was fragmented to a size of ~400 bp using a Covaris S2 ultrasonicator (Covaris Inc., MA, US). The resulting FFPE DNA fragments were processed with the NEBNext Ultra II End Repair/dA-Tailing Module (NEB, Cat. no. E7546 L), followed by adapter ligation using the NEBNext Ultra II Ligation Module (NEB, Cat. no. E7595 L). The DNA library was purified using SPRIselect beads (Beckman, Cat. no. B23318) and underwent bisulfite conversion with the NEBNext Enzymatic Methyl-seq Conversion Module (NEB, Cat. no. E7125 L). After 8–14 cycles of PCR amplification, the library was purified again using SPRIselect beads (0.9×) to remove contaminants. Sequencing was performed on a NovaSeq 6000 platform, generating 2 × 150-bp paired-end reads for downstream analysis.

**Comparison and Evaluation of EM-seq and WGBS based on FFPE Samples.**

To evaluate the performance of the EM-seq technology (NEB) in FFPE sample library preparation, a pilot study was conducted using FFPE samples from two patients. EM-seq and whole-genome bisulfite sequencing (WGBS) libraries were prepared in parallel and sequenced using the same platform. A comparative analysis was then performed to assess key performance metrics, including genome coverage, CT conversion rates, genome alignment rates, duplication rates, data usability, and average methylation levels of genomic elements. This comparison provides insights into the relative effectiveness and efficiency of EM-seq and WGBS, informing the optimization of library preparation methods for future experiments.

**RNA-sequencing**

Total RNA (200–800 ng) extracted from each sample was depleted of ribosomal RNA using the Ribo-off rRNA Depletion Kit (Vazyme, Cat. no. N406) according to the manufacturer's instructions, followed by purification with 2.2× VAHTS RNA Clean Beads (Vazyme, Cat. no. N412-01). The purified RNA was then used for cDNA library preparation with the VAHTS Universal V8 RNA-seq Library Prep Kit for Illumina (Vazyme, Cat. no. NR605), without RNA fragmentation. Adapter-ligated products were amplified by PCR for 10–14 cycles, and the resulting RNA-seq library was purified twice with SPRIselect beads (0.9×) to remove impurities. Library quality was assessed, and sequencing was performed on a NovaSeq 6000 platform, yielding 2 × 150-bp paired-end reads for downstream analysis.

**DNA Methylation Analysis**

During initial data preprocessing, DNA reads in fastq format were quality-filtered using Trimmomatic (v0.36) ^[1]^ and aligned to the hg19 reference genome (including the 48,502 bp λDNA genome) using Bismark (v0.23.1) ^[2]^ with specific parameters (-N 1). PCR duplicates were removed with Bismark, and overlapping regions were clipped using BamUtil (v1.0.15) to enhance data reliability. The resulting clean bam files were used for downstream analyses. In the downstream analysis, CpG sites with ≥3 informative reads and located outside ENCODE blacklist regions were retained. Biscuit (v0.1.4) ^[3]^ was used to calculate average methylation levels in specific genomic regions, while MethylKit (v1.24.0) ^[4]^ computed genome-wide average methylation levels in 10-kb bins. Average methylation levels of gene promoters were used for cell composition deconvolution with EPISCORE (v0.9.5) ^[5]^ to estimate proportions of epithelial, endothelial, fibroblast, and immune cells. Additionally, 848,170 methylation haplotype blocks (MHBs) were identified, and methylation haplotype load (MHL) and average methylation fraction (AMF) were calculated based on previous studies ^[6]^. MHBs with >10% NA values were excluded, and the remaining NAs were imputed using the median value for each MHB. Functional annotations of MHBs were explored using rGREAT (v2.0.2) ^[7]^.

**Copy Number Analysis**

Copy number variation (CNV) was analyzed using QDNAseq (v1.34.0) ^[8]^ and ichorCNA (v0.3.21.3) ^[9]^. QDNAseq identified CNVs at the 10-kb bin level using the hg19 reference genome, with regions showing an absolute log_2_ copy number ratio ≥0.585 (equivalent to log_2_(1.5)) classified as losses or gains. Recurrent arm-level and focal CNV segments were identified using GISTIC2.0 ^[10]^. IchorCNA analysis included three steps: (1) readCounter analysis of HMMcopy-generated WIG files with 500-kb bins covering all autosomes, filtering out reads with mapping quality <20; (2) copy number analysis using parameters including --normal, --ploidy, --txnE, --txnStrength, --lambda, and other defaults; and (3) calculation of the weighted genome instability index (wGII) by assessing the proportion of CNV length relative to total genome length, providing a measure of genome-wide instability.

**Transcriptomic Analysis**

During initial data preprocessing, RNA-seq raw data in fastq format were quality-filtered using fastp (v0.23.2) ^[11]^ and aligned to the GRCh37 reference genome using the STAR aligner (v2.7.8a) ^[12]^, with gene expression levels quantified using GRCh37 annotations. In the downstream analysis, transcriptome data were log_2_-transformed into transcripts per million (TPM). Differential expression and log_2_-transformed counts per million (CPM) were analyzed using DESeq2 (v1.38.3) ^[13]^. Gene set enrichment analysis (GSEA) was performed using GSVA (v1.46.0) ^[14]^, with single-sample GSEA (ssGSEA) calculating enrichment scores for specified gene sets. Gene Ontology (GO) analysis was conducted using ClusterProfiler (v4.6.2) ^[15]^, with GO terms and enriched gene sets considered significant at *p* < 0.05. Tumor microenvironment (TME) classification was performed using TMEclassifier in IOBR (v0.998) ^[16, 17]^, and cell abundance deconvolution was estimated using CIBERSORTx ^[18]^, with single-cell RNA data as a reference. Additionally, a curated list of anoikis-related genes (ARGs) from a previous study ^[19]^ was used for differential expression analysis in both bulk RNA-seq data from the discovery cohort and single-cell RNA-seq data from public datasets. Finally, pathway and transcription factor activity inference analysis were performed using OmnipathR and decoupleR ^[20]^.

**RNA-based Mutation Calling**

Single-nucleotide mutations were identified from the RNA-seq data via the GATK pipeline. Insertions and deletions (indels) were not included in this study because of potential technical challenges associated with their identification from RNA-seq data. Specifically, STAR v2.7.8a was employed to align the raw RNA reads to the hg19 human genome assembly, and PICARD tools were utilized to mark duplicate reads. Subsequently, GATK tools, namely SplitNCigarReads, BaseRecalibrator, and ApplyBQSR, were applied to reformat certain alignments spanning introns and to correct the base quality scores. Ultimately, HaplotypeCaller was used for variant calling. The resulting Variant Call Format (VCF) files were annotated using SnpEff, followed by a filtering process to assess their potential impact on proteins. Initially, only single-nucleotide variants (SNVs) annotated with a HIGH or MODERATE impact by SnpEff were retained, while SNVs located at splice-site genomic regions were excluded. Second, mutations with an rs ID in the dbSNP database were removed. Third, only mutations with a quality score exceeding 100 and a Fisher Strand score (FS) below 30.0 were included. Finally, RNA-based mutations were visualized using the Maftools R package.

**Single‑cell RNA Sequencing Analysis**

Normalized scRNA-seq data from two T1HG UCB patients ^[21]^ were analyzed using Seurat (v5.0.0) ^[22]^. The top 2,000 variable features were identified for each dataset and integrated using the "FindIntegrationAnchors" and "IntegrateData" functions. Principal component analysis (PCA) was then applied to reduce data dimensionality, with the first 30 principal components used for UMAP-based dimensionality reduction. Cell clusters were identified using the "FindClusters" function at a resolution of 0.8, allowing for the delineation of distinct cellular populations. Cell annotations were assigned based on established methods from the original literature to ensure accurate classification and interpretation of the scRNA-seq data.

**Derivation of T1HG-UCBguider**

**Feature extraction.** To identify biomarkers associated with tumor anoikis, apoptosis, and immune suppression, we analyzed gene expression profiles using GeneSelectR (v1.0.1) (<https://github.com/dzhakparov/GeneSelectR>) with a normalized vector of 284 gene expression features. The input data, combined with multi-omic subtypes from 88 patients, were used to model binary labels. Feature significance was assessed using four machine learning techniques—univariate Cox, Random Forest, LASSO, and Boruta—which reduced the feature dimensions from 284 to 141. GeneSelectR inherently incorporates bootstrap-based stability assessment by evaluating the consistency of selected features across multiple resampled datasets; only features with high selection frequency were retained for downstream analysis. A two-sided Wilcoxon rank-sum test further refined the features to those with significant expression differences (*p* < 0.05). GeneSelectR also facilitated GO enrichment analysis and clustering of GO terms, retaining genes enriched in pathways related to metastasis, apoptosis, and immune response. This process identified 18 mRNA biomarkers. For MHB biomarker extraction, GO enrichment analysis was conducted based on the 18 mRNA biomarkers to select pathways containing multiple genes. MHBs with differential methylation Δ values compared to T1HG2 and inversely correlated with gene expression log_2_FoldChange values were identified. Additional GO enrichment analysis was performed on annotated genes of these MHBs, selecting those corresponding to pathways containing two or more mRNA biomarker genes. A two-sided Wilcoxon rank-sum test filtered MHBs with significant differences between subtypes (*p* < 0.05), identifying 7 final MHB biomarkers. To ensure consistency, MHL and AMF were computed for MHB biomarkers. mRNA biomarker values included log_2_-transformed TPM, normalized GSVA scores, and normalized ssGSEA scores, enhancing the robustness of the selection process.

**Dataset and model training.** The discovery cohort of 88 patients was used to train machine learning classification models. To obtain unbiased performance estimates and avoid overfitting during hyperparameter tuning, a nested cross-validation (CV) strategy was employed. In the inner loop of the nested CV, hyperparameters were optimized through five-fold cross-validation to maximize the area under the receiver operating characteristic (ROC AUC) curve. Hyperparameters were optimized through five-fold cross-validation to maximize the area under the receiver operating characteristic (ROC AUC) curve. After optimization, the remaining parameters were finalized by retraining the models with the optimal hyperparameters on the full training dataset. Considering the cost constraints of multi-omic detection technologies in clinical settings, we prioritized single-omics approaches that maintain model performance. We combined 18 mRNA markers and 7 MHB markers with four clinical indicators (age, gender, tumor stage, and grade) to construct two sub-models: clin_RNA and clin_MHB. These sub-models were integrated into a comprehensive model, T1HG-UCBguider, which combines molecular markers and clinical parameters to enhance diagnostic and predictive accuracy for T1HG UCB.

**T1HG-UCBguider architecture.** The machine learning framework was developed in Python (version 3.7.4) using key libraries including scikit-learn (v0.21.2), numpy (v1.16.4), scipy (v1.3), and pandas (v0.24.2) within a conda environment. Each sub-model was structured as an ensemble of three scikit-learn pipelines, with the final prediction calculated as the mean of scores from these pipelines. Each pipeline included four steps: feature elimination for collinearity, k-best feature selection, feature scaling, and classification. Initially, features with a Pearson correlation coefficient >0.8 were removed, retaining only the feature most strongly correlated with the response variable. Features not ranking within the top k based on ANOVA F-value relative to the binary response were excluded. Remaining features were scaled using z-score normalization. For classification, the first pipeline used logistic regression with elastic net regularization and the SAGA solver (C parameters ranging from 10^-3^ to 10^3^ , L1 ratios from 0.1 to 1); the second pipeline used a support vector classifier with radial basis function, sigmoid, or linear kernels (gamma parameters from 10^-9^ to 10^-2^ , C parameters from 10^-3^ to 10^3^ ); and the third pipeline used a random forest approach with 5 to 100 estimators, maximum features ranging from 5% to 70% of the total, and minimum samples per split between 2 and 15. Hyperparameters were optimized through a randomized 1,000-step five-fold cross-validation search to maximize the ROC AUC.

**Multi-cohort validation of T1HG-UCBguider.** The models were evaluated in the validation cohort by assessing ROC curves and AUCs. To provide internal validation and quantify the uncertainty of model performance, we used bootstrapping with 2,000 resamples on the discovery cohort via the roc() function from the R package pROC with the argument ci=TRUE. This generated 95% confidence intervals for all reported AUC values, accounting for sampling variability. The "surv_cutpoint" function in survminer (v0.4.9) was used to determine the threshold for categorizing patients into high-risk (HR) and low-risk groups based on model prediction scores. Survival curves were generated to evaluate the models' performance in predicting recurrence, progression, and response to BCG treatment. For the integrated model, validation incorporated data from the external validation cohort (*n* = 59), which included paired EM-seq and RNA-seq datasets of tumor tissues from T1HG UCB patients treated with BCG. For the clin_MHB sub-model, validation incorporated data from an external cohort and two sources: validation cohort 1 (*n* = 48) included whole-genome DNA methylation sequencing of tumor tissues from T1HG patients prepared in-house, while validation cohort 2 (*n* = 27) used preoperative urine methylation sequencing data from a published study. For the clin_RNA sub-model, comparisons were made with the PI-23 genes model across multiple cohorts, as well as a combined RNA cohort incorporating data from these cohorts. Validation cohort 3 (*n* = 55) was established using RNA extracted from FFPE tumor tissues in a prospective cohort study initiated in 2022, with 37 patients undergoing BCG treatment. Additionally, three publicly available transcriptome sequencing datasets were used: validation cohort 4 (*n* = 71) from GSE154261, with only 18 mRNA markers used due to incomplete clinical data; validation cohort 5 (*n* = 460) from E-MTAB-4321, an early UROMOL cohort dataset with 78 T1HG UCB patients; and validation cohort 6 (*n* = 103) from GSE13507. The training dataset for the PI-23 genes model, with sequencing data obtained via microarray technology.

**Reproduction of 23-Gene Prognostic Index Model**

We used the hazard ratio values in Extended Data Table 3 provided in the article ^[23]^ to generate the Cox regression model formula for the PI-23 genes. The 23 genes include *FOXM1*, *CKAP2L*, *E2F1*, *XBP1*, *LIN9*, *CSF1*, *S100A9*, *PCLAF*, *SPP1*, *SREBF2*, *E2F2*, *S100A8*, *MYB*, *CD24*, *FANCD2*, *SH3TC2*, *MASTL*, *NAMPT*, *SOD2*, *EHMT1*, *HOXB3*, MNT, and *TSC1*. However, for the genes missing from the log_2_CPM expression matrix provided in the article as well as in other cohorts' expression matrix datasets (*PCLAF*, NAMPT, or *CD24*), we set the model coefficients of those genes to 0.

**Supplementary References**

[1] Bolger AM, Lohse M, Usadel B. Trimmomatic: A flexible trimmer for illumina sequence data. Bioinformatics, 2014, 30: 2114-2120

[2] Krueger F, Andrews SR. Bismark: A flexible aligner and methylation caller for bisulfite-seq applications. Bioinformatics, 2011, 27: 1571-1572

[3] Zhou W, Johnson BK, Morrison J, et al. Biscuit: An efficient, standards-compliant tool suite for simultaneous genetic and epigenetic inference in bulk and single-cell studies. Nucleic Acids Res, 2024, 52: e32

[4] Akalin A, Kormaksson M, Li S, et al. Methylkit: A comprehensive r package for the analysis of genome-wide DNA methylation profiles. Genome Biol, 2012, 13: R87

[5] Teschendorff AE, Zhu T, Breeze CE, et al. Episcore: Cell type deconvolution of bulk tissue DNA methylomes from single-cell rna-seq data. Genome Biol, 2020, 21: 221

[6] Guo S, Diep D, Plongthongkum N, et al. Identification of methylation haplotype blocks aids in deconvolution of heterogeneous tissue samples and tumor tissue-of-origin mapping from plasma DNA. Nat Genet, 2017, 49: 635-642

[7] Gu Z, Hubschmann D. Rgreat: An r/bioconductor package for functional enrichment on genomic regions. Bioinformatics, 2023, 39:

[8] Scheinin I, Sie D, Bengtsson H, et al. DNA copy number analysis of fresh and formalin-fixed specimens by shallow whole-genome sequencing with identification and exclusion of problematic regions in the genome assembly. Genome Res, 2014, 24: 2022-2032

[9] Adalsteinsson VA, Ha G, Freeman SS, et al. Scalable whole-exome sequencing of cell-free DNA reveals high concordance with metastatic tumors. Nat Commun, 2017, 8: 1324

[10] Mermel CH, Schumacher SE, Hill B, et al. Gistic2.0 facilitates sensitive and confident localization of the targets of focal somatic copy-number alteration in human cancers. Genome Biol, 2011, 12: R41

[11] Chen S, Zhou Y, Chen Y, et al. Fastp: An ultra-fast all-in-one fastq preprocessor. Bioinformatics, 2018, 34: i884-i890

[12] Riker RR, Stone PC, Jr., May T, et al. Initial bispectral index may identify patients who will awaken during therapeutic hypothermia after cardiac arrest: A retrospective pilot study. Resuscitation, 2013, 84: 794-797

[13] Love MI, Huber W, Anders S. Moderated estimation of fold change and dispersion for rna-seq data with deseq2. Genome Biol, 2014, 15: 550

[14] Hanzelmann S, Castelo R, Guinney J. Gsva: Gene set variation analysis for microarray and rna-seq data. BMC Bioinformatics, 2013, 14: 7

[15] Yu G, Wang LG, Han Y, et al. Clusterprofiler: An r package for comparing biological themes among gene clusters. OMICS, 2012, 16: 284-287

[16] Zeng D, Li M, Zhou R, et al. Tumor microenvironment characterization in gastric cancer identifies prognostic and immunotherapeutically relevant gene signatures. Cancer Immunol Res, 2019, 7: 737-750

[17] Zeng D, Ye Z, Shen R, et al. Iobr: Multi-omics immuno-oncology biological research to decode tumor microenvironment and signatures. Front Immunol, 2021, 12: 687975

[18] Newman AM, Steen CB, Liu CL, et al. Determining cell type abundance and expression from bulk tissues with digital cytometry. Nat Biotechnol, 2019, 37: 773-782

[19] Xie T, Peng S, Liu S, et al. Multi-cohort validation of ascore: An anoikis-based prognostic signature for predicting disease progression and immunotherapy response in bladder cancer. Mol Cancer, 2024, 23: 30

[20] Badia IMP, Velez Santiago J, Braunger J, et al. Decoupler: Ensemble of computational methods to infer biological activities from omics data. Bioinform Adv, 2022, 2: vbac016

[21] Chen Z, Zhou L, Liu L, et al. Single-cell rna sequencing highlights the role of inflammatory cancer-associated fibroblasts in bladder urothelial carcinoma. Nat Commun, 2020, 11: 5077

[22] Hao Y, Hao S, Andersen-Nissen E, et al. Integrated analysis of multimodal single-cell data. Cell, 2021, 184: 3573-3587 e3529

[23] Kim SK, Byun YJ, Park SH, et al. A 23-gene prognostic index predicts progression and bacillus calmette-guerin response in non-muscle-invasive bladder cancer. Eur Urol, 2024, 85: 400-402

**Supplementary Figure Legends**

**FIGURE S1 | Identification and validation of two distinct molecular subtypes in T1 high-grade bladder cancer with significant prognostic implications.**

(**A**) Clinical characteristics of the discovery cohort (*n* = 88) and external validation cohort (*n* = 59). The cohorts are balanced for age, gender, and recurrence/progression status, with BCG treatment administered in 37.5% (discovery) and 100% (validation) of patients. (**B**) Unsupervised consensus clustering analysis identifies two robust molecular subtypes: T1HG1 (*n* = 39) and T1HG2 (*n* = 49). Hierarchical clustering based on multi-omic profiles reveals clear separation between subtypes, supported by a high average silhouette width (0.88), indicating strong cluster cohesion and separation. (**C**) Kaplan-Meier survival curves in the discovery cohort demonstrate significant differences in clinical outcomes between T1HG1 and T1HG2 subtypes. The number at risk is shown below each curve. (**D**) External validation in an independent cohort confirms the prognostic power of the T1HG classification. The log-rank test was used to compare the Kaplan–Meier survival curves (C and D). A p-value < 0.05 is considered statistically significant. Statistical analysis was carried out using R (v4.2.1).

**FIGURE S2 | Multi-omic profiling reveals distinct mutational, genomic, DNA methylation, and transcriptomic landscapes between T1HG1 and T1HG2 subtypes of T1 high-grade bladder cancer.**

(**A**) Mutation burden and spectrum analysis. Top panel: Boxplot showing the percentage of mutations by nucleotide substitution type (C>T, T>C, etc.) across all samples, with C>T transitions being the most prevalent. Bottom panel: Stacked bar plot of mutation frequencies across individual samples. (**B**) Copy number alteration (CNA) profiles in T1HG1 and T1HG2. GISTIC 2.0 analysis identifies recurrent gains (red) and losses (blue) across the genome. (**C**) Genomic instability assessment via weighted genome instability index (wGII). (**D**) Annotation distribution of 1505 prognostic methylation haplotype blocks (MHBs). (**E**) Gene Ontology (GO) enrichment analysis of differentially methylated genes associated with prognosis. Hypo-methylated genes (red) are enriched in processes related to cell cycle regulation. Hyper-methylated genes (blue) are enriched in metabolic processes and immune response. (**F**) Functional enrichment of significantly upregulated (left) and downregulated (right) genes in T1HG1 vs. T1HG2. Upregulated genes in T1HG1 are enriched in developmental pathways, whereas downregulated genes are enriched in immune-related functions (leukocyte migration, T-cell activation, cytokine signaling). (**G**) Comparative histogram of DNA damage response (DDR) genes with statistically significant differential expression, highlighting significantly lower expression of base excision repair (BER) pathway genes (CCNO and PARP3) in T1HG1 compared to T1HG2 (|log_2_FC| > 0.5). Each data point represents an individual sample, the two-tailed unpaired Mann-Whitney U test (C) was used for statistical analysis in R (v4.2.1). A p-value < 0.05 is considered statistically significant.

**FIGURE S3 | Immune cell-specific gene signatures and macrophage polarization profiles in T1HG1 and T1HG2 subtypes.**

(**A**) Hazard ratios (HR) for immune-related genes associated with prognosis in T cells (left) and macrophages (right), derived from multivariable Cox regression analysis. Lower HR values suggest protective effects of these genes against disease progression. (**B**) UMAP plot of single-cell RNA sequencing (scRNA-seq) data from two T1 high-grade bladder cancer tumors, showing 33 distinct cell clusters. (**C**) Proportions of M0 (naïve) and M2 (pro-tumorigenic) macrophage subsets in the discovery cohort (left) and external validation cohort (right). Each data point represents an individual sample, the two-tailed unpaired Mann-Whitney U test (C and D) was used for statistical analysis in R (v4.2.1). A p-value < 0.05 is considered statistically significant.

**FIGURE S4 | NQO1 drives anoikis resistance and promotes tumor progression in T1 high-grade bladder cancer.**

(**A**) Relative tumor anoikis scores in the discovery (top left) and external validation (top right) cohorts. Single-sample Gene Set Enrichment Analysis (ssGSEA) of upregulated anti-anoikis genes (up ARGs) and downregulated ARGs (down ARGs) in the discovery cohort (middle) and external validation cohort (bottom). T1HG1 tumors exhibit significantly higher anoikis resistance compared to T1HG2. (**B**) Log_2_ fold change of ARGs significantly upregulated or downregulated in the external validation cohort. (**C**) Kaplan-Meier survival curve from The Cancer Genome Atlas (TCGA) cohort stratified by NQO1 expression level (high vs. low). Patients with high NQO1 mRNA expression have significantly worse recurrence-free survival. (**D**) mRNA expression of NQO1 in the external validation cohort. T1HG1 tumors show significantly higher NQO1 expression than T1HG2, consistent with its expression in the discovery cohort. (**E**) Validation of Nqo1 knockdown efficiency in MB49 mouse bladder cancer cells using qRT-PCR. Four independent shRNA constructs (shNQO1-1 to -4) significantly reduce NQO1 mRNA levels compared to control (shControl), with shNQO1-1 and -4 showing the most potent suppression and selected for subsequent experiments. (**F**) In vivo tumor growth kinetics in subcutaneous models (5 C57BL mice per group) using MB49 cells transduced with shNqo1 or shControl. The log-rank test was used to compare the Kaplan–Meier survival curves (C). Each data point represents an individual sample, the two-tailed unpaired Mann-Whitney U test (A and D), two-tailed unpaired Student's t-test (E), was used for statistical analysis in R (v4.2.1). A p-value < 0.05 is considered statistically significant.

**FIGURE S5 | Skullcapflavone II and dicoumarol exhibit potent anti-tumor activity in vitro and in vivo, with enhanced efficacy when combined with cisplatin.**

(**A**) Dose-response inhibition profiles of Skullcapflavone II (SFII) in human bladder cancer cell lines T24, J82, and murine MB49 cells treated with increasing concentrations of SFII. Heatmaps show percentage inhibition at each dose, revealing concentration-dependent efficacy. (**B**) Schematic diagram of the in vivo study design, with treatment of cisplatin (3 mg/kg, i.p.) and/or dicoumarol (50 mg/kg, i.p.) in the subcutaneous model. (**C**) Tumor volume growth curves over 24 days. The combination of dicoumarol and cisplatin results in significantly suppressed tumor growth compared to cisplatin or vehicle control. (**D**) Final tumor weights at endpoint confirm dramatic reduction in the dicoumarol and combination group. (**E**) Body weight changes over time during treatment. No significant weight loss was observed in any group. (**F**) Histopathological analysis of excised tumors by H&E and PAS staining revealed minimal changes across all groups, indicating that the NQO1 inhibitor and its combination with cisplatin did not cause additional adverse reactions such as kidney damage. The vehicle group showed mild fibroblast proliferation and scant inflammatory cell infiltration, with no evident basement membrane thickening. Compared to the vehicle, the cisplatin group exhibited focal necrosis of renal tubular epithelial cells but no significant fibroblast proliferation or other abnormalities. In the SFII group, occasional thickening of the Bowman's capsule basement membrane was observed, without notable inflammation. The dicoumarol group displayed mild tubular epithelial edema, vacuolar degeneration, and rare necrosis, but no fibrosis. In the cisplatin + SFII group, occasional proteinaceous casts and mild tubular edema were noted, along with rare Bowman's capsule basement membrane thickening; no inflammatory infiltrates were detected. The cisplatin + dicoumarol group showed mild tubular atrophy, slight epithelial edema, and occasional tubular basement membrane thickening, but no significant inflammation. Collectively, these findings indicate that none of the treatments induced severe nephrotoxicity under the experimental conditions. Data are shown as mean ± SEM. The p-values were calculated using one-way (D) and two-way (C) ANOVA, followed by Tukey's post hoc test. A p-value < 0.05 is considered statistically significant. Statistical analysis was carried out using GraphPad Prism (v8.0.2).

**FIGURE S6 | Cell type proportion analysis of macrophages in the MB49 subcutaneous model following NQO1 knockdown.**

The relative proportions of macrophage subpopulations were estimated from RNA-seq data using CIBERSORTx deconvolution analysis. Tumor tissues were harvested from C57BL/6 male mice (n=5 per group) subcutaneously injected with MB49 cells stably transduced with either shControl or shNqo1 lentiviruses. Each data point represents an individual sample, the two-tailed unpaired Mann-Whitney U test (C and D) was used for statistical analysis in R (v4.2.1). A p-value < 0.05 is considered statistically significant.

**FIGURE S7 | Integrated multi-omic analysis identifies a robust prognostic signature for T1 high-grade bladder cancer based on mRNA and DNA methylation markers.**

(**A**) Heatmap displaying the clustering of GO terms for feature genes following the dimension reduction of 284 mRNA features utilizing GeneSelectR. (**B**) GO enrichment analysis of 18 mRNA markers. (**C**) Volcano plot displaying significantly affected mRNA markers (|logFC| > 0.5 and adjusted p-value < 0.05), marked in red dots (upregulated genes in T1HG1) and blue dots (downregulated genes in T1HG1), with the numbers of significantly upregulated and downregulated genes indicated below the plot. (**D**) Quadrant chart showing significantly affected MHB markers (|logFC| > 0.5 and |ΔMHL| > 0.05), with the numbers of significantly induced and repressed genes indicated below the plot. (**E**) GO enrichment analysis of 7 MHB markers. (**F**) Dot plot illustrating the expression levels of 18 mRNA markers and 7 MHB markers across cell types based on a single cell RNA-seq dataset. (**G**) AUC curves were generated to predict progression and recurrence in the discovery cohort (left) and the BCG-treated subset (right). (**H**) RFS and PFS curves were generated for risk stratification in the discovery cohort (left panels) and the BCG-treated subset (right panels). AUCs with 95% CI were labeled in all ROC plots (G). The log-rank test was used to compare the Kaplan–Meier survival curves (H). A p-value < 0.05 is considered statistically significant. Statistical analysis was carried out using R (v4.2.1).

**FIGURE S8 | Multi-cohort validation and comparative analysis of clin_RNA vs. 23-Gene Prognostic Index model.**

(**A-B**) AUC (left), RFS (middle), and PFS (right) curves were generated for performance benchmarking in risk stratification of T1HG patients from the validation cohort 3 (*n* = 55; prospective) (A), validation cohort 4 (*n* = 71) (B). (**C-F**) AUC (left), RFS (middle), and PFS (right) curves were generated for performance benchmarking in risk stratification of NMIBC patients from validation cohort 6 (*n* = 103; training set of the 23-Gene Prognostic Index) (C), validation cohort 5 (*n* = 460; UROMOL cohort) (D), the combined RNA cohort (*n* = 836), the BCG-treated subset of the combined RNA cohort (*n* = 320) (E-F). To fairly compare the performance of the two models, the combined RNA cohort included all available RNA data from all cohorts, including the training set of 23-Gene Prognostic Index. AUCs with 95% CI were labeled in all ROC plots (A-F). The log-rank test was used to compare the Kaplan–Meier survival curves (A-F). A p-value < 0.05 is considered statistically significant. Statistical analysis was carried out using R (v4.2.1).

**FIGURE S9 | Ethnicity-stratified evaluation of T1HG-UCBguider performance.** (**A**) Subgroup Cox regression analyses, model discrimination metrics, and interaction tests across ethnic populations. Hazard ratios (HRs) with 95% CI, concordance index (C-index), and time-dependent area under the curve (AUC) are presented separately for Caucasian and Asian cohorts in the overall T1HG population and in patients receiving BCG therapy. Interaction p-values were derived from Cox models including an interaction term between risk score and ethnicity to assess potential effect modification. (**B**) Kaplan–Meier curves of progression-free survival (PFS) and recurrence-free survival (RFS) stratified by the T1HG-UCBguider risk groups within each ethnic subgroup. In the overall cohort, the model effectively stratified patients into distinct risk groups in both Caucasian and Asian populations. In the BCG-treated subgroup, significant risk stratification was observed in Asian patients, while limited discrimination in Caucasian patients may be attributable to the small sample size. Multivariate Cox regression analysis was performed to analyze the relative risk of progression and recurrence, and the significance of the interaction between model score and ethnicity was assessed using the Wald test (A). The log-rank test was used to compare the Kaplan–Meier survival curves (B). A p-value < 0.05 is considered statistically significant. Statistical analysis was carried out using R (v4.2.1).

**FIGURE S10 | Calibration curves of the T1HG-UCBguider models in all validation cohorts.** Calibration plots depict the agreement between predicted probabilities and observed outcomes (PFS and RFS) for T1HG-UCBguider models across various validation cohorts. Each panel corresponds to a specific cohort and model type.

**FIGURE S11 | User-friendly web interface of T1HG-UCBguider and demonstration of output results from a test file.** This integrated visualization enables intuitive interpretation of multi-omic model prognostic predictions for T1HG bladder cancer.

**FIGURE S12 | Experimental technique evaluation and data quality control of EM-seq and RNA-seq for FFPE samples.**

(**A**) Comparison of library and sequencing metrics for EM-seq and WGBS libraries using two FFPE bladder cancer samples. Metrics were calculated using Bismark, Samtools, and Picard. (**B**) Comparison of DNA methylation level for EM-seq and WGBS libraries in different genome elements. (**C**) PCA plot of individual samples in the discovery cohort. (**D**) Gene expression heatmap of housekeeping genes in the discovery cohort. (WGBS: Whole Genome Bisulfite Sequencing; EM-seq: Enzymatic Methylation sequencing; Expected coverage: the number of bases sequenced/total bases in the hg19 reference; % Mapped: percentage of reads aligned to the reference genome (hg19+controls); % Dups: percentage of reads marked as duplicate by Picard MarkDuplicates; % Usable: percentage of the set of Proper-pair, MapQ 10+, primary, nonduplicate reads used in methylation calling (SAMtools view -F 0xF00 -q 10); PCA: Principal Component Analysis).

**Supplementary Figures**

**
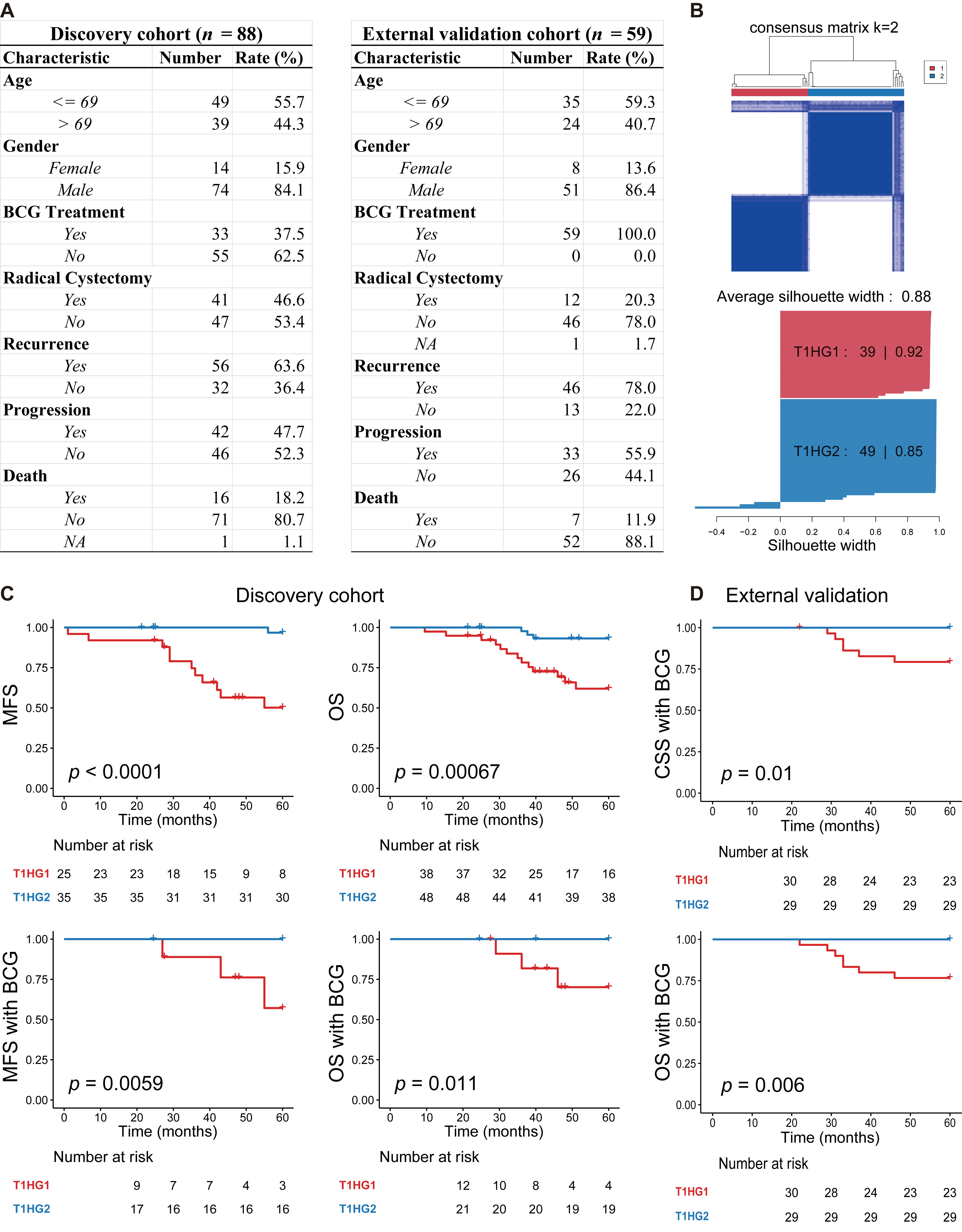
**

**FIGURE S1 | Identification and validation of two distinct molecular subtypes in T1 high-grade bladder cancer with significant prognostic implications.**

**
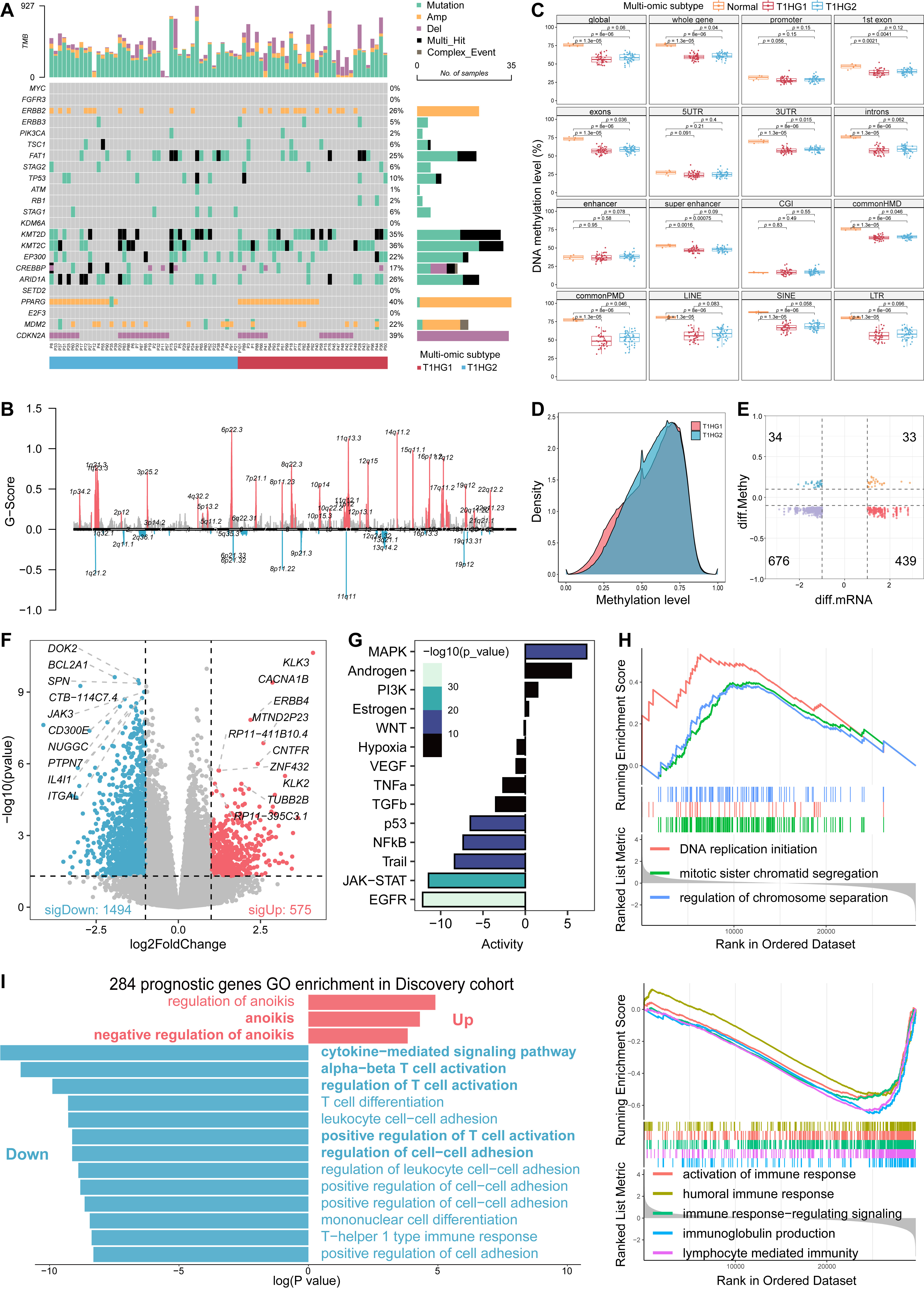
**

**FIGURE S2 | Multi-omic profiling reveals distinct mutational, genomic, DNA methylation, and transcriptomic landscapes between T1HG1 and T1HG2 subtypes of T1 high-grade bladder cancer.**

**
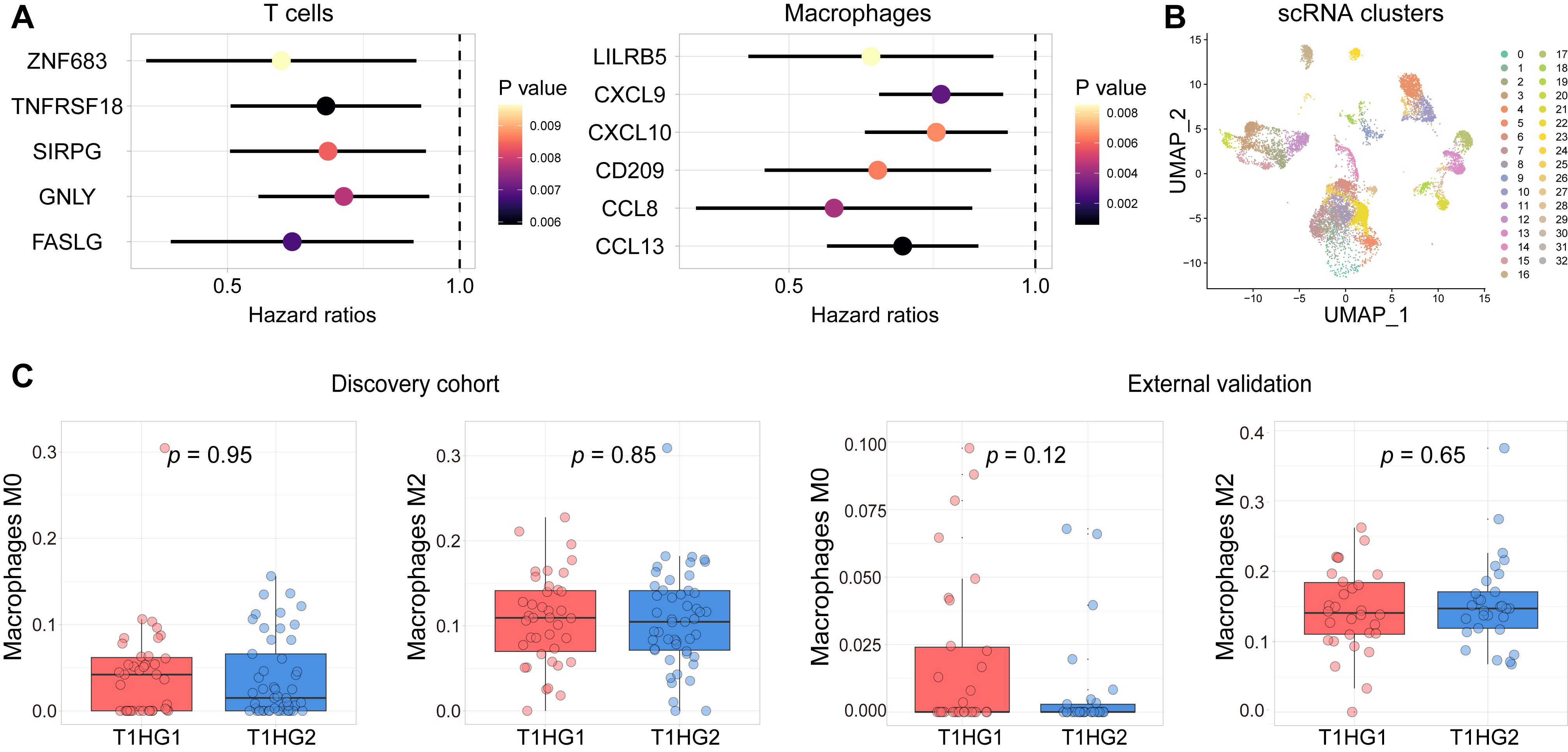
**

**FIGURE S3 | Immune cell-specific gene signatures and macrophage polarization profiles in T1HG1 and T1HG2 subtypes.**

**
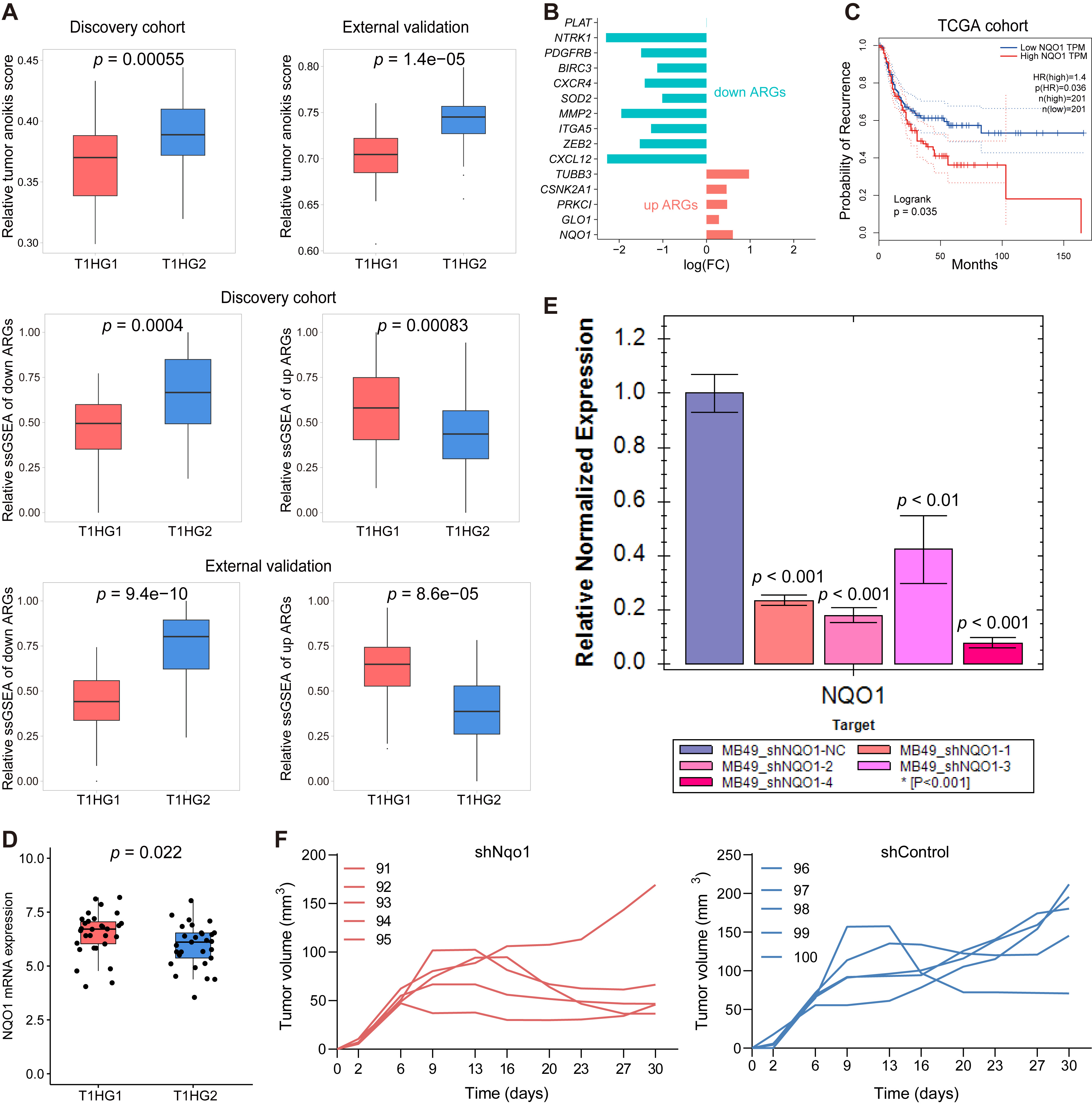
**

**FIGURE S4 | NQO1 drives anoikis resistance and promotes tumor progression in T1 high-grade bladder cancer.**

**
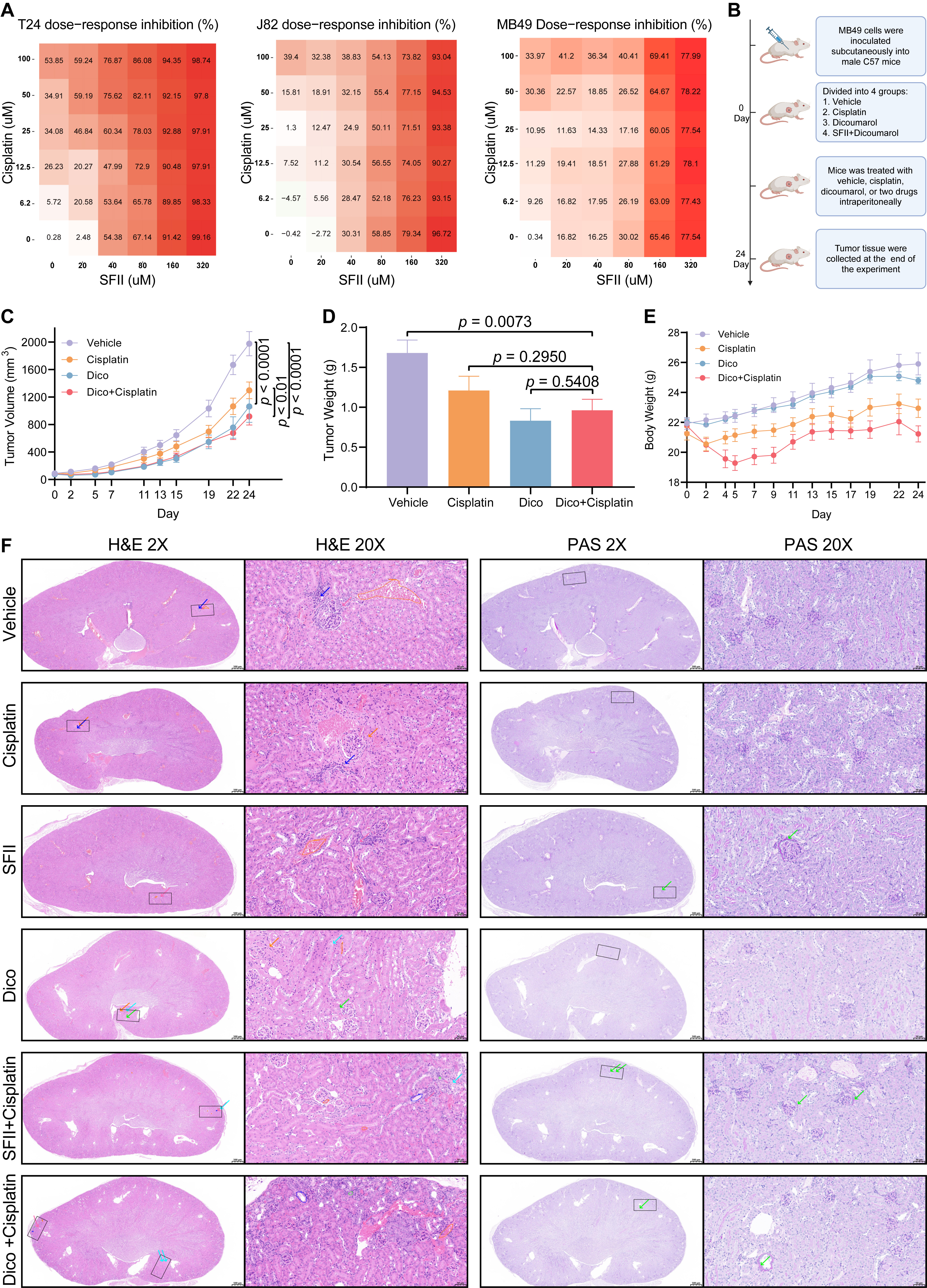
**

**FIGURE S5 | Skullcapflavone II and dicoumarol exhibit potent anti-tumor activity in vitro and in vivo, with enhanced efficacy when combined with cisplatin.**

**
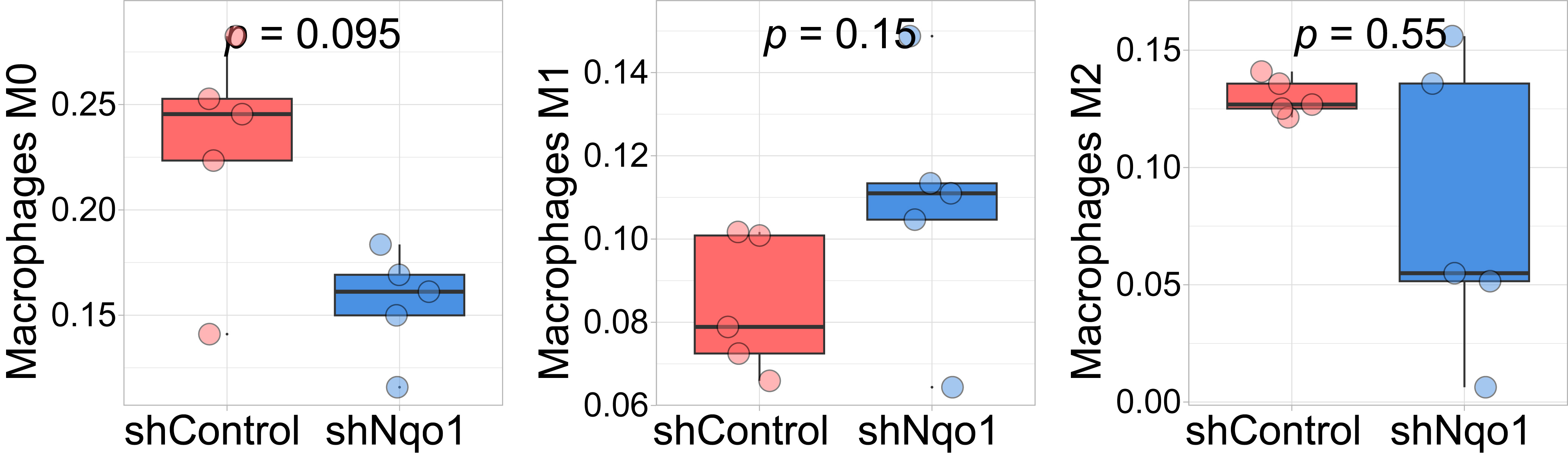
**

**FIGURE S6 | Cell type proportion analysis of macrophages in the MB49 subcutaneous model following NQO1 knockdown.**

**
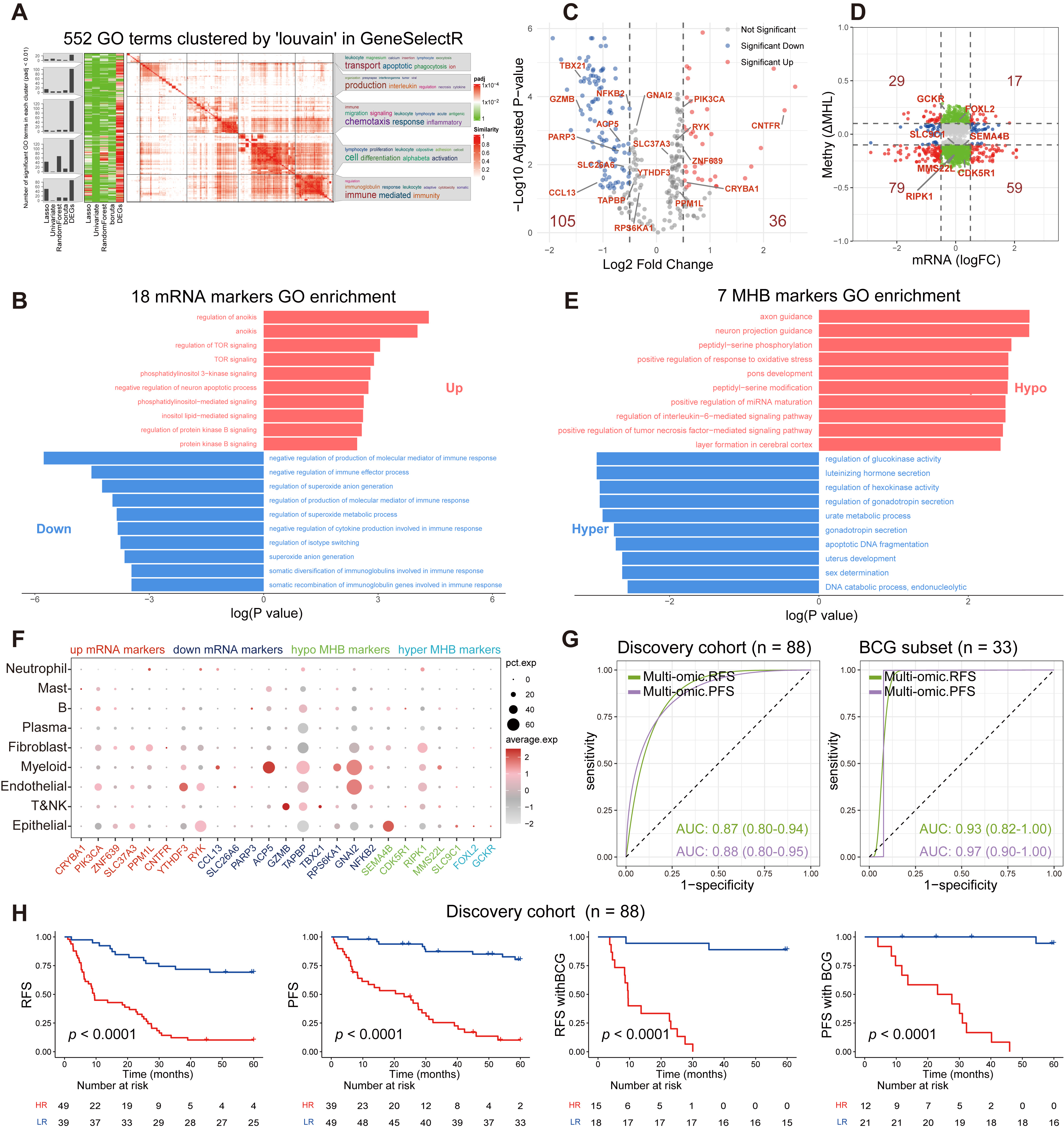
**

**FIGURE S7 | Integrated multi-omic analysis identifies a robust prognostic signature for T1 high-grade bladder cancer based on mRNA and DNA methylation markers.**

**
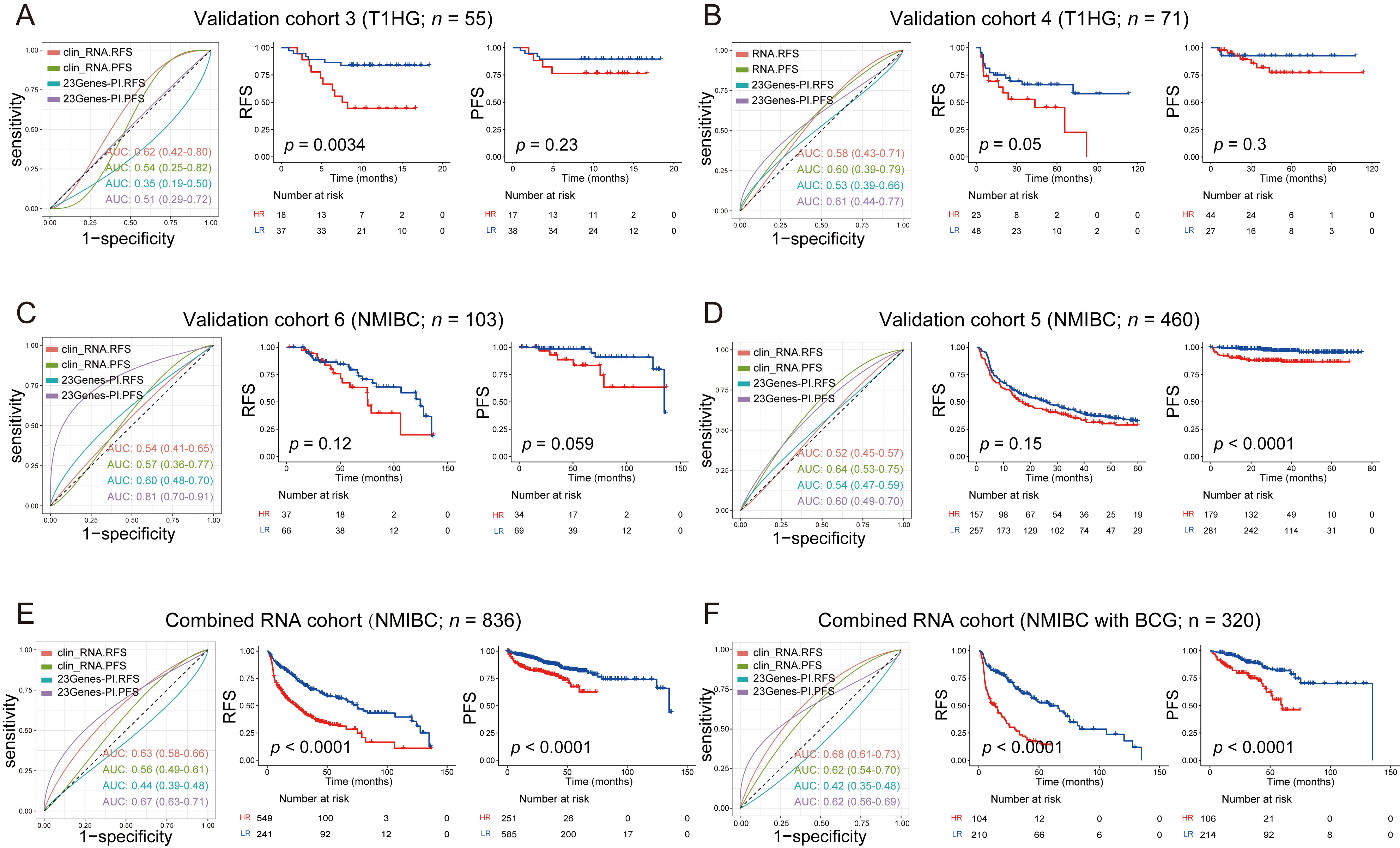
**

**FIGURE S8 | Multi-cohort validation and comparative analysis of clin_RNA vs. 23-Gene Prognostic Index model.**

**
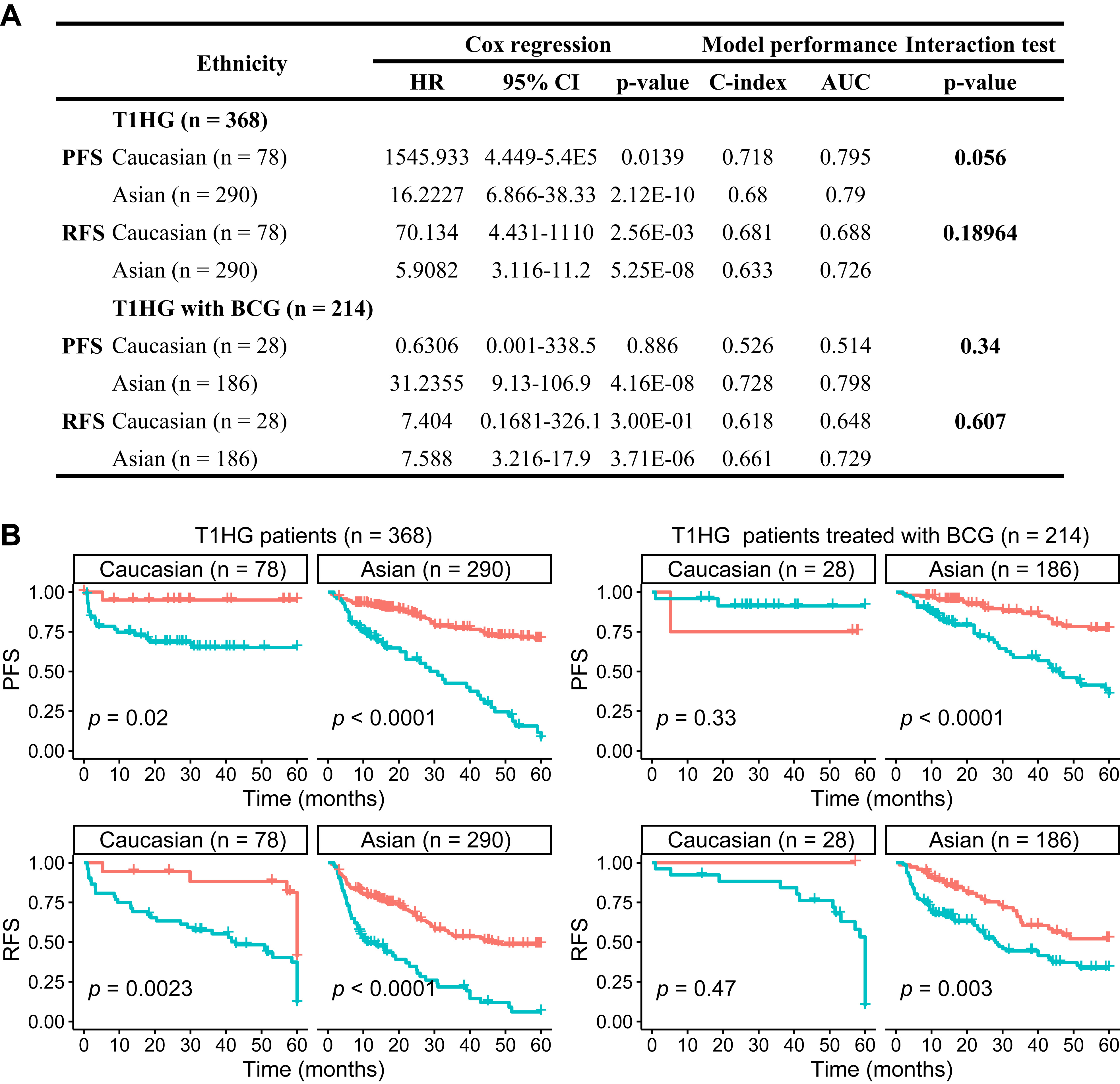
**

**FIGURE S9 | Ethnicity-stratified evaluation of T1HG-UCBguider performance.**

**
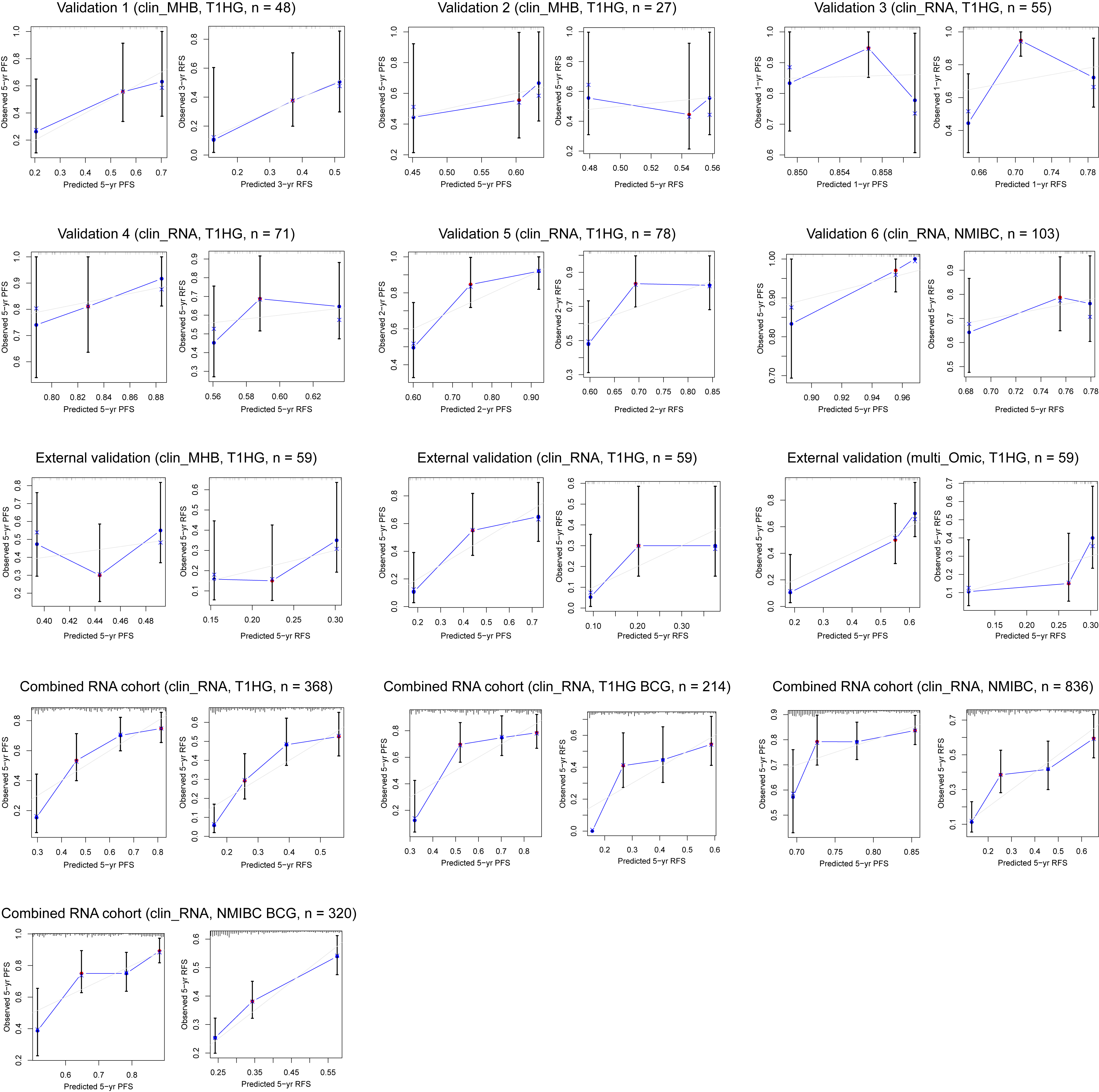
**

**FIGURE S10 | Calibration curves of the T1HG-UCBguider models in all validation cohorts.**

**
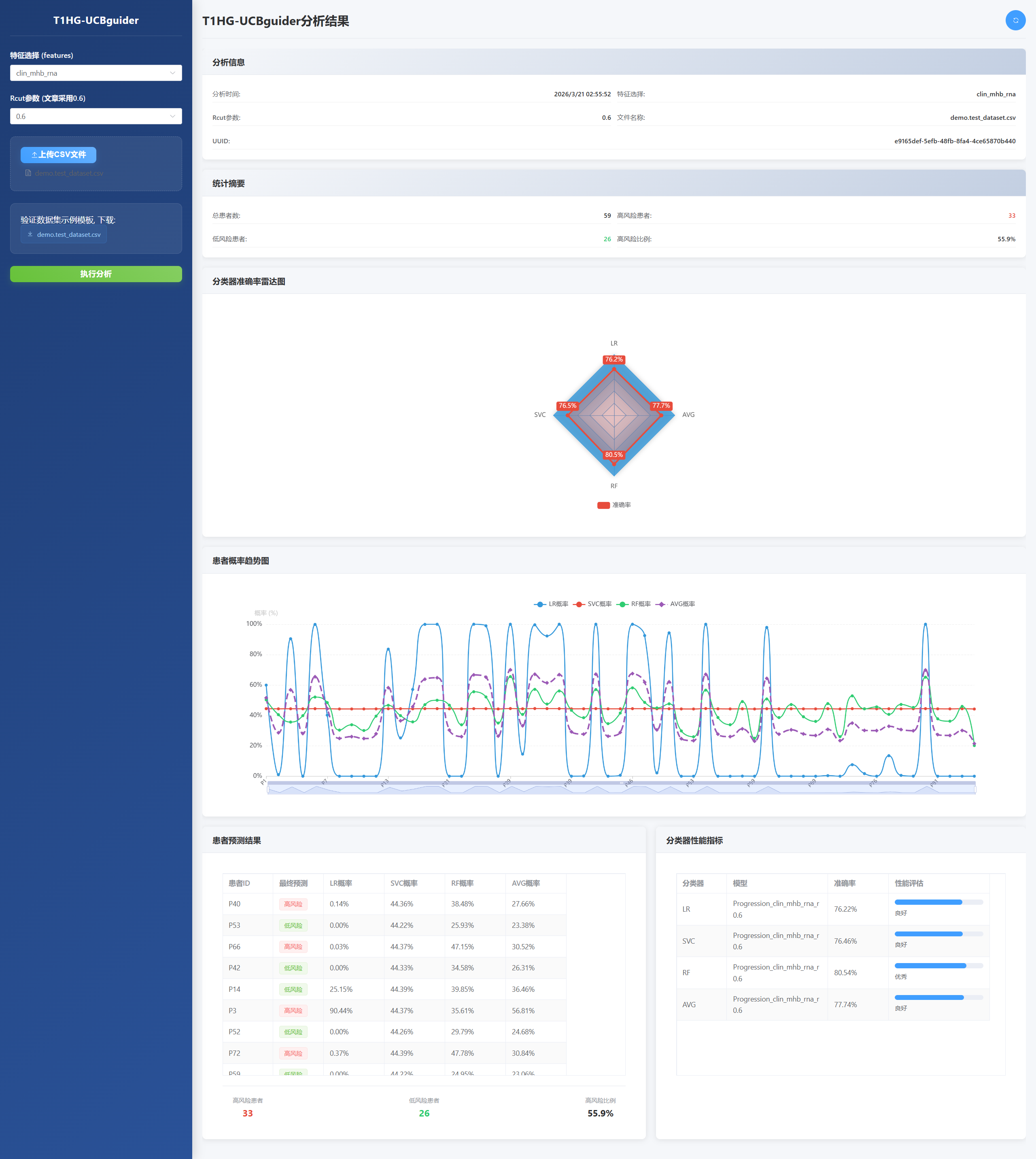
**

**FIGURE S11 | User-friendly web interface of T1HG-UCBguider and demonstration of output results from a test file.**

**
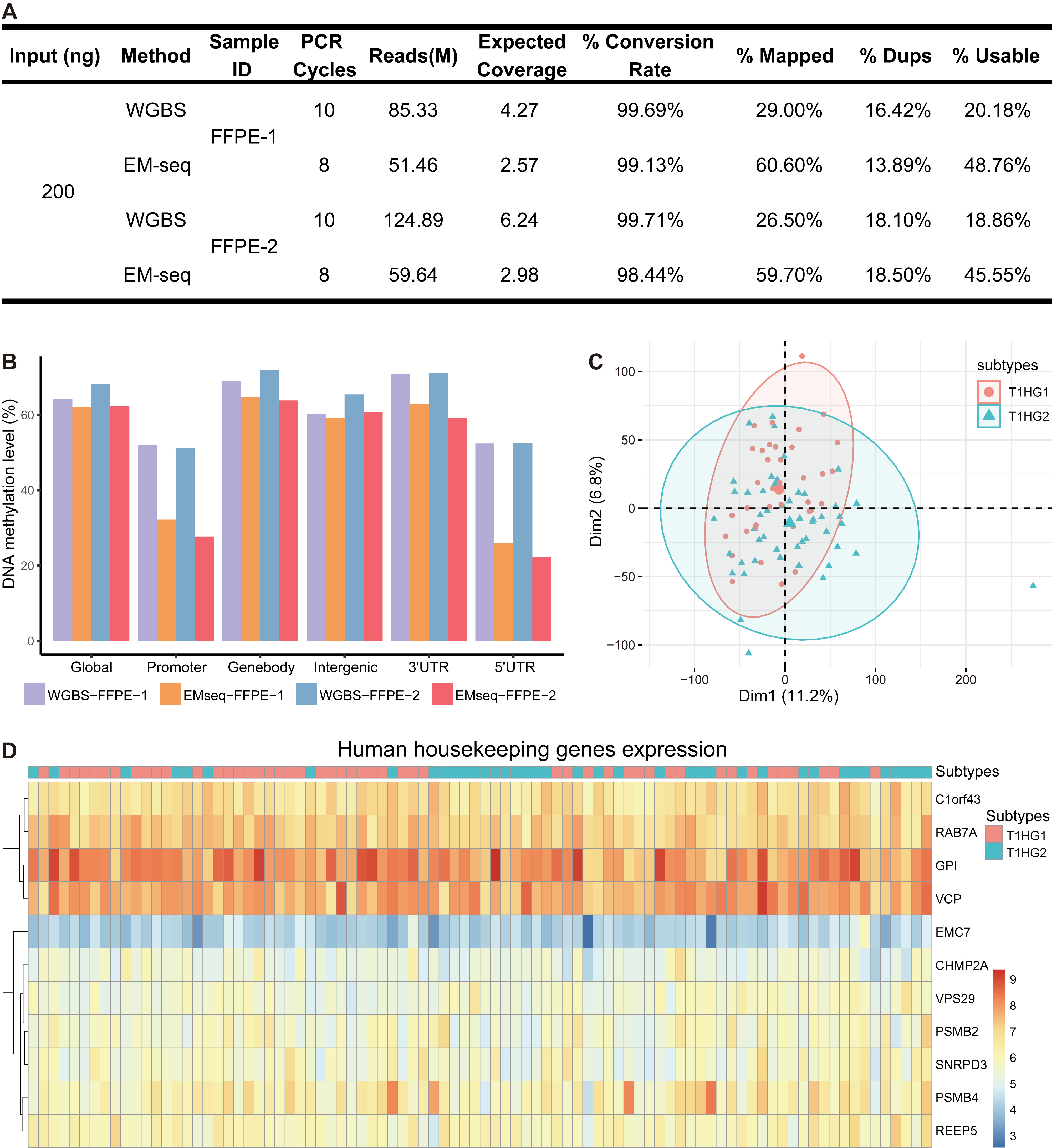
**

**FIGURE S12 | Experimental technique evaluation and data quality control of EM-seq and RNA-seq for FFPE samples.**

**Supplementary Table**

**Table S1.** **Comparative performance of T1HG-UCBguider and 23-gene Prognostic Index in various cohorts.**

Abbreviations: FFPE, Formalin-Fixed and Paraffin-Embedded; EM-seq, Enzymatic Methylation-sequencing; NMIBC, Non-Muscle-Invasive Bladder Cancer; PFS, Progression-Free Survival; RFS, Recurrence-Free Survival; BCG, Bacillus Calmette-Guérin; AUC, Area Under Curves; PI-23genes, 23-gene Prognostic Index; NA, not available.

**Table S2. Antibodies used for multiplex immunofluorescence staining.**

| **Antibody** | **Host Species** | **Manufacturer** | **Catalog No.** | **Dilution** |
| --- | --- | --- | --- | --- |
| anti-NQO1 | Rabbit | Proteintech | 11451-1-AP | 1:500 |
| anti-pan-CK | Mouse | Servicebio | GB122053-50 | 1:500 |
| anti-CD68 | Mouse | Servicebio | GB123150-50 | 1:500 |
| anti-CD8 | Mouse | Servicebio | GB12068-50 | 1:1500 |
| anti-CXCL9 | Rabbit | Abcam | ab290643 | 1:200 |
| anti-CXCR3 | Rabbit | Servicebio | GB11659-50 | 1:1000 |
| anti-PD-1 | Mouse | Servicebio | GB12338-50 | 1:1000 |
| anti-SPP1 | Rabbit | Servicebio | GB11500-50 | 1:500 |

**Table S3. Plasma concentration of Skullcapflavone II in mice (60 mg/kg, i.p., sample from 5 mice per time point, N = 25)**

| Time (h) | Drug concentration (ng**·**mL^-1^) | | | | | | | | | | Mean  (ng**·**mL^-1^) | SD  (ng**·**mL^-1^) | CV% |
| --- | --- | --- | --- | --- | --- | --- | --- | --- | --- | --- | --- | --- | --- |
|  | Me521 | Me522 | Me523 | Me524 | Me525 | NA | NA | NA | NA | NA |  |  |  |
| 0 | 0.000 | 0.000 | 0.000 | 0.000 | 0.000 | NS | NS | NS | NS | NS | 0.0000 | 0.0000 | NA |
| Time (h) | Durg concentration (ng**·**mL^-1^) | | | | | | | | | | Mean  (ng**·**mL^-1^) | SD  (ng**·**mL^-1^) | CV% |
|  | Me501 | Me502 | Me503 | Me504 | Me505 | Me506 | Me507 | Me508 | Me509 | Me510 |  |  |  |
| 0.25 | 10063.122 | 10036.614 | 8385.824 | 12831.247 | 7879.919 | NS | NS | NS | NS | NS | 9839.3452 | 1936.0102 | 19.6762 |
| 0.5 | NS | NS | NS | NS | NS | 2297.128 | 2640.746 | 1877.746 | 2488.416 | 3761.564 | 2613.1200 | 702.8271 | 26.8961 |
| Time (h) | Drug concentration (ng**·**mL^-1^) | | | | | | | | | | Mean  (ng**·**mL^-1^) | SD  (ng**·**mL^-1^) | CV% |
|  | Me511 | Me512 | Me513 | Me514 | Me515 | Me516 | Me517 | Me518 | Me519 | Me520 |  |  |  |
| 1 | 291.614 | 313.247 | 111.008 | 101.572 | 526.572 | NS | NS | NS | NS | NS | 268.8026 | 174.5035 | 64.9188 |
| 2 | NS | NS | NS | NS | NS | 15.359 | 7.334 | 18.022 | 40.099 | 19.567 | 20.0762 | 12.1450 | 60.4947 |
| Time (h) | Drug concentration (ng**·**mL^-1^) | | | | | | | | | | Mean  (ng**·**mL^-1^) | SD  (ng**·**mL^-1^) | CV% |
|  | Me521 | Me522 | Me523 | Me524 | Me525 | Me501 | Me502 | Me503 | Me504 | Me505 |  |  |  |
| 4 | 9.972 | 25.516 | 193.630 | 8.754 | 13.555 | NS | NS | NS | NS | NS | 50.2854 | 80.4058 | 159.8988 |
| 8 | NS | NS | NS | NS | NS | 6.394 | 1.905 | 7.530 | 4.269 | 1.905 | 4.4006 | 2.5613 | 58.2024 |
| Time (h) | Drug concentration (ng**·**mL^-1^) | | | | | | | | | | Mean  (ng**·**mL^-1^) | SD  (ng**·**mL^-1^) | CV% |
|  | Me506 | Me507 | Me508 | Me509 | Me510 | Me511 | Me512 | Me513 | Me514 | Me515 |  |  |  |
| 12 | 2.072 | 1.096 | 3.155 | 1.305 | BQL | NS | NS | NS | NS | NS | 1.9070 | 0.9318 | 48.8630 |
| 24 | NS | NS | NS | NS | NS | 1.013 | BQL | BQL | BQL | BQL | 1.0130 | NA | NA |
| Time (h) | Drug concentration (ng**·**mL^-1^) | | | | | | | | | | Mean  (ng**·**mL^-1^) | SD  (ng**·**mL^-1^) | CV% |
|  | Me516 | Me517 | Me518 | Me519 | Me520 | NA | NA | NA | NA | NA |  |  |  |
| 48 | BQL | BQL | BQL | BQL | BQL | NS | NS | NS | NS | NS | NA | NA | NA |

**Note:** Linear range: 1–15000 ng/mL; LLOQ (Lower Limit of Quantification): 1 ng/mL; NS (No Sample): No sample; NA (Not Available): Not applicable; BQL (Below Quantificable Limit): Below the quantitative limit; Me523 at 4 h is an outlier.

**Table S4. Average pharmacokinetic parameters of Skullcapflavone II in plasma (60 mg/kg, i.p., sample from 5 mice per time point, N = 25)**

| **Compound** | **No_points_lambda_z** | **t_1/2_**  **(h)** | **T_max_**  **(h)** | **C_max_**  **(ng/mL)** | **AUC_0~t_**  **(h*ng/mL)** | **AUC_0~∞_obs**  **(h*ng/mL)** | **AUC_%Extrap_obs**  **(%)** | **AUC_0~t__D_obs**  **(h*kg*ng/mL/mg)** | **MRT_0~t_ (h)** |
| --- | --- | --- | --- | --- | --- | --- | --- | --- | --- |
| Skullcapflavone Ⅱ | 3.000 | 8.373 | 0.250 | 9839.345 | 3861.265 | 3873.502 | 0.316 | 64.354 | 0.632 |

.
